# Effects of Physical Exercise Combined with Nutritional Supplements on Aging Brain Related Structures and Functions: A Systematic Review

**DOI:** 10.3389/fnagi.2016.00161

**Published:** 2016-07-06

**Authors:** Alexandra Schättin, Kilian Baur, Jan Stutz, Peter Wolf, Eling D. de Bruin

**Affiliations:** ^1^Department of Health Sciences and Technology, Institute of Human Movement Sciences and Sport, Swiss Federal Institute of Technology (ETH Zurich)Zurich, Switzerland; ^2^Sensory-Motor Systems Lab, Department of Health Sciences and Technology, Swiss Federal Institute of Technology (ETH Zurich)Zurich, Switzerland

**Keywords:** nutritional supplementation, nutrition, physical exercise, brain function, brain metabolism, aging

## Abstract

Age-related decline in gray and white brain matter goes together with cognitive depletion. To influence cognitive functioning in elderly, several types of physical exercise and nutritional intervention have been performed. This paper systematically reviews the potential additive and complementary effects of nutrition/nutritional supplements and physical exercise on cognition. The search strategy was developed for EMBASE, Medline, PubMed, Cochrane, CINAHL, and PsycInfo databases and focused on the research question: “Is the combination of physical exercise with nutrition/nutritional supplementation more effective than nutrition/nutritional supplementation or physical exercise alone in effecting on brain structure, metabolism, and/or function?” Both mammalian and human studies were included. In humans, randomized controlled trials that evaluated the effects of nutrition/nutritional supplements and physical exercise on cognitive functioning and associated parameters in healthy elderly (>65 years) were included. The systematic search included English and German language literature without any limitation of publication date. The search strategy yielded a total of 3129 references of which 67 studies met the inclusion criteria; 43 human and 24 mammalian, mainly rodent, studies. Three out of 43 human studies investigated a nutrition/physical exercise combination and reported no additive effects. In rodent studies, additive effects were found for docosahexaenoic acid supplementation when combined with physical exercise. Although feasible combinations of physical exercise/nutritional supplements are available for influencing the brain, only a few studies evaluated which possible combinations of nutrition/nutritional supplementation and physical exercise might have an effect on brain structure, metabolism and/or function. The reason for no clear effects of combinatory approaches in humans might be explained by the misfit between the combinations of nutritional methods with the physical interventions in the sense that they were not selected on sharing of similar neuronal mechanisms. Based on the results from this systematic review, future human studies should focus on the combined effect of docosahexaenoic acid supplementation and physical exercise that contains elements of (motor) learning.

## Introduction

Thirty percent of people aged 65 and older living in the community experience at least one fall per year, and this proportion increases markedly with age (Tromp et al., 2001). The elevated incidence of falls in elderly is only one of the many physical dysfunctions that may be encountered with advanced age (Iosa et al., 2014). Elderly experience a reduction in walking speed, an increased variability in step timing, a decline in gait stability, and are compromised in their learning ability (Cai et al., 2014; Iosa et al., 2014). These reductions in movement functionality often develop already in midlife (Tomey and Sowers, 2009) and have been described as age-related deteriorations in physical functioning (Pichierri et al., 2011). Physical functioning can be defined as the ability to conduct activities that are required for independent living and that may affect quality of life, such as walking and climbing stairs (Painter et al., 1999; Pichierri et al., 2011). Limitations in physical functioning have been associated with depression, increased risk for falls and injuries, reduced quality of life, increased health care costs, and mortality (Tomey and Sowers, 2009). In the near future, the number of elderly people suffering from physical dysfunctions will increase due to demographic changes (Kluge et al., 2014).

Various factors have been proposed causing the decline in physical functioning in the older population. Loss of muscle mass, strength, and degradation of joints have been associated with gait instability and falls (Mithal et al., 2013; Iosa et al., 2014). In addition to reductions in the musculoskeletal system, impairments in vision, reaction time, and balance play an important role (Iosa et al., 2014). However, worsening of the sensorimotor system is probably not the only cause explaining deteriorations in physical functioning: reduction of cognitive functions is believed to play a significant role as well (Cai et al., 2014; Iosa et al., 2014). Cognitive functions are “…any mental process that involves symbolic operations—e.g., perception, memory, creation of imagery, and thinking…” (Concise Dictionary of Modern Medicine, 2002).

A decrease in cognitive performance in old age is predominant in most individuals. Aging associated cognitive decline has a prevalence rate of 28% for people from 65 to 84 years (Scafato et al., 2010). Another 17% of the population investigated (*n* = 4785) showed objective evidence of cognitive decline without cognitive complaints, which sums up to a total of 45% of people showing some kind of cognitive impairment without dementia. Cognitive aging is characterized by mental decline (Cotman et al., 2007), memory impairments (Gunning-Dixon et al., 2009; van Praag, 2009; Cai et al., 2014), decreased learning ability (van Praag, 2009; Cai et al., 2014), greater anxiety and poorer attention (Cai et al., 2014), slower processing speed (Gunning-Dixon et al., 2009; Clouston et al., 2013; Cai et al., 2014), and reduction of executive skills (Gunning-Dixon et al., 2009). Other studies showed that healthy participants and participants with cognitive impairment or subclinical cerebrovascular lesions had reduced gait stability and postural control during dual-task walking suggesting that cognitive abilities affect gait performance (Pichierri et al., 2011; Choi et al., 2012; Iosa et al., 2014).

Studies investigating cognitive aging support the idea that neuroanatomical changes, such as loss of brain tissue and cortical disconnections, might explain the poorer cognitive performance of healthy elderly (Colcombe et al., 2003; Resnick et al., 2003; Raz et al., 2005; Gunning-Dixon et al., 2009). Age-dependent structural changes include loss of gray matter volume (frontal and temporal lobes), vulnerability of prefrontal white matter, loss of microstructural white matter integrity, and decrease in hippocampus and cerebellum volumes (Raz et al., 2005; Gunning-Dixon et al., 2009). For example, frontal lobe white matter has been proposed to mediate the association of age and performance in tasks assessing executive skills and memory (Brickman et al., 2006). Structural alterations might partially account for observed age-dependent declines in cognition (Gunning-Dixon et al., 2009). In addition to the neuroanatomical changes, neurochemical processes change during the course of aging (Mora, 2013). In rodents, for example, an age-dependent decrease of neurotrophic factors, such as the brain-derived neurotrophic factor (BDNF), might contribute to age-related cognitive impairments (Gooney et al., 2004; Adlard et al., 2005; Mora et al., 2007).

**Figure 1:**
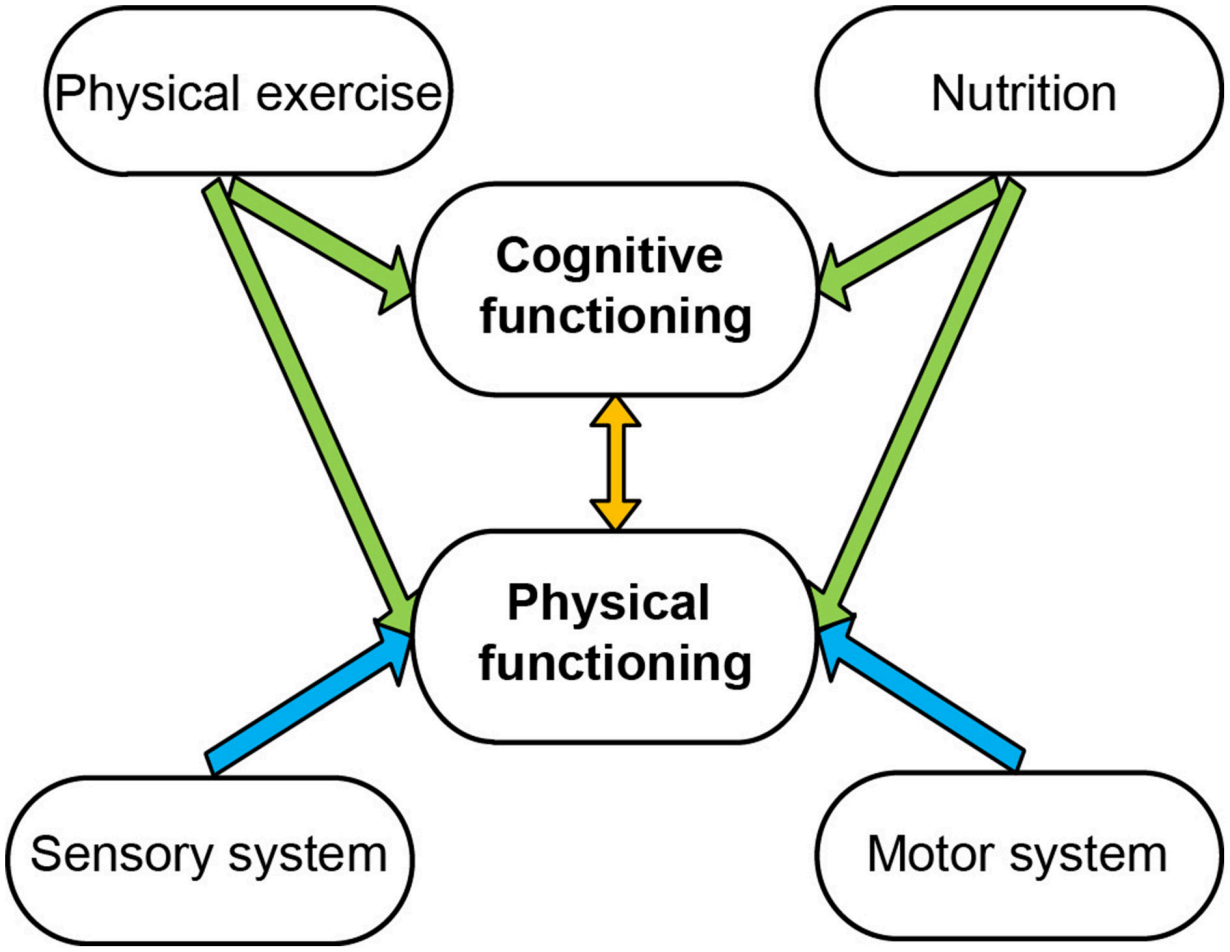
**Interaction of cognitive and physical functioning**. Physical and cognitive functioning influences each other (orange arrow). In turn, both can be influenced by physical exercise and nutrition (green arrows). The motor and sensory system regulates physical functioning (blue arrows).

Since cognitive decline potentially threatens independence and quality of life of older adults, prevention and treatment of cognitive impairment in the elderly has assumed increasing importance (Williams and Kemper, 2010). Two factors that may positively effect on cognition are physical activity (Gomez-Pinilla and Hillman, 2013) and nutritional supplementation (Gómez-Pinilla, 2008; Figure 1). Physical exercise has been described to be the most effective way to maintain a healthy body and mind (van Praag, 2009). Physical exercise lowers blood pressure, increases sensitivity to insulin, contributes to weight loss, delays age-related cognitive decline, improves learning and memory, and reduces the risk of neurodegeneration (Shephard and Balady, 1999; Cotman and Berchtold, 2002). The proposed mechanisms by which physical exercise affects cognition revolve around changes in neurotransmitters, neurotrophins, and vasculature (Cotman et al., 2007). Neurogenesis in the hippocampus is associated with improved cognition, and the strongest neurogenic stimulus seems to be physical exercise (van Praag, 2009). Moreover, physical exercise appears to affect properties of dendritic spines, to enhance long term potentiation, to influence brain vasculature through the actions of insulin like growth factor (IGF) and vascular endothelial growth factor, and to affect BDNF which plays an essential role in synaptic plasticity and cell genesis, growth, and survival (van Praag, 2009).

Nutrition and nutritional supplements may also exhibit positive effects on brain health (van Praag, 2009). Studies showed that caloric restriction (CR) and nutritional supplements such as fish oil, teas, fruits, folate, spices, and vitamins have the potential to positively effect on cognitive functioning (Gómez-Pinilla, 2008). Investigations on the effects of nutrition on brain function have usually focused on neuroprotective properties of nutritional supplements (van Praag, 2009). Recent studies focused on underlying mechanisms like neuronal signaling (van Praag, 2009). In fact, nutritional supplementation and CR seem to affect similar cellular and molecular pathways as physical exercise (van Praag, 2009).

From the foregoing, the assumption that physical exercise and nutrition could have additive effects on brain structures and functions that may result in greater benefits on cognition for combinatory interventions seems justified (Gómez-Pinilla, 2011). “Additive” means when two interventions are combined intendedly (physical exercise and nutrition are an integral part of one intervention) to enhance effects and “complementary” in case each intervention stands by itself. Recent studies indicate that exercise is capable of boosting the health effects of certain diets and that selected dietary factors may have the capacity to complement the effects of exercise (Gómez-Pinilla, 2011). However, existing reviews on these effects are either narrative, or, when being performed systematically, are limited in the sense that they focus on the isolated effect of either physical exercise or nutrition (Gómez-Pinilla, 2008; van Praag, 2009; Voss et al., 2013). Which combination of selected dietary factors possibly best should be added to physical exercise for additive effects of exercise on cognition in humans remains, therefore, indefinite. To the best of our knowledge, a systematic review focusing on the possible additive effects of physical exercise and nutrition/nutritional supplementation on the elderly brain has not been performed. Therefore, a systematic review was performed on the effect of combined physical and nutritional interventions with the aim of clarifying the relationship between the type of combined intervention and the effects of such an intervention on brain related structure and function in both mammalian and human studies. The following research question guided this systematic review: “Is the combination of physical exercise with nutritional supplementation more effective than nutritional supplementation or physical exercise alone in effecting on brain structure, metabolism, and/or function”?

## Method

### Data sources and searches

A search strategy was developed in collaboration with a librarian from the Medical Library of the University of Zurich. The search period covered all years from the inception to October, 2015, and included EMBASE, Medline, PubMed, Cochrane, CINAHL, and PsycInfo. Searches were undertaken using MeSH headings and text words including the following main terms for the population: *aged, elder, placental mammals, human, rat, mouse, mice, mammal, mammalia*; for nutritional intervention: *diet supplementation, diet therapy, protein intake, dietary intake, diet, protein, nutrient, mineral, vitamin, supplementation, supplement, additive, intake, therapy, treatment*; for physical exercise: *resistance training, physical, activity, exercise, fitness, strength, training*, and for the outcome of interest: *cognition, executive function, memory, nervous system development, nerve cell plasticity, angiogenesis, neurogenesis, synaptogenesis, neuroplasticity, brain structure, spine density, function, structure, neurotransmitter, vascular endothelial growth factor, insulin like growth factor, brain, nerve*. Furthermore, the bibliographies of all eligible articles and related reviews, as well as recent conference proceedings, were checked through hand searching. To ensure the clarity and transparency of reporting, the PRISMA guidelines (Moher et al., 2010) were followed.

### Selection criteria

Both studies with mammalians and humans were considered for this review. From mammalian research knowledge about the effects on molecular, cellular, and neural circuit levels and how these may impact cognitive function can be gained (Voss et al., 2013; Gutchess, 2014). Higher-level cognition effects and influences on macro- and systems-level change in the central nervous system can be evaluated in human studies (Voss et al., 2013; Gutchess, 2014). The search strategy included “elderly over the age of 65 years” and “older mammalians.” Interventions that focused on physical exercise and nutritional supplementation or the combination of both were considered. Study outcomes were determined on brain structure, -function, and -metabolism levels. Randomized controlled trials (RCT), the most rigorous way of determining whether a cause-effect relation exists between treatment and outcome (Sibbald and Roland, 1998), were primarily included. Because well-designed observational studies have been shown to provide results similar to randomized controlled trials (Song and Chung, 2010) case control trials were also included. In addition, reviews on our topic written in English or German with no year restriction were considered for discussion.

### Selection process

The first step was the removal of duplicate citations. Afterwards two reviewers (JS, AS) determined which studies should be included by independently screening of title, abstract, and keywords. A priori set inclusion and exclusion criteria were applied to the articles (Table 1). An article was eligible, if the investigator examined complementary or additive effects of physical exercise and nutritional interventions on cognitive functions in humans and mammalians and/or associated brain parameters in mammalians. Only longitudinal studies were included that carried out an intervention. Interventions that considered aerobe, strength, and/or coordination training were defined as physical exercise. Nutritional interventions were those that considered nutritional supplementation. Studies using pharmacological supplementation or that focused on physical outcomes were excluded. Subsequently, the results from the screening were discussed to exclude any differences in the inclusion decisions. Full text reading of the remaining literature yielded the final list of papers. Studies were included assessing brain structure (e.g., gray and white matter), brain metabolism (e.g., neurotrophic factors), and brain function (e.g., cognitive test batteries) in healthy elderly.

**Table 1:** **List of inclusion and exclusion criteria**.

**Area**	**Inclusion criteria**	**Exclusion criteria**
Population	Older (>65 years) adults and old mammalians	Patients with neurodegenerative diseases
Intervention	Nutritional supplementation, brain food, physical exercise, exercise trainings, physical activity	Pharmacological interventions
Outcome	Neurogenesis, synaptic plasticity, brain structure, spine density, angiogenesis, growth factors, neurotransmitter, neurotrophins, cognitive function	Physical benefits
Study type	Randomized controlled and case control trails	Methodological, theoretical, review, and discussion papers
Language	English and German	All other languages
Year	All years	–

### Data extraction and data synthesis

The included studies were sub-divided into human and mammalian studies. Each of these classes was then further subdivided into three groups: Physical exercise, nutritional intervention, and studies that investigated a combination of physical exercise and nutritional intervention. In this way, comparisons between different treatments were easier to track. A purpose adjusted individualized data extraction form from Wright et al. (2007) was used to collect data from single studies. The extraction of the data included (1) reference information: author and date; (2) characteristics of study population: number of participants, gender, age, genetics (mammalians); (3) characteristics of physical exercise intervention: type of exercise, frequency, and duration; (4) characteristics of nutritional intervention: diet or nutritional supplement, amount of intake, and duration; (5) characteristics of outcomes: outcome measures and results. The data is presented in the results section as a descriptive summary of the studies and their results. Furthermore, a qualitative synthesis of the studies was executed. A meta-analysis was not performed due to the high heterogeneity of intervention types and outcome variables among the studies.

### Quality appraisal

Quality evaluation of the studies was done by reporting potential sources of bias (Harris et al., 2013). For critical quality appraisal, the purpose-adjusted Downs and Black checklist for randomized and non-randomized studies of health care interventions was used (Downs and Black, 1998). The quality checklist consisted of 27 items having a theoretical maximum score of 32 points. The checklist scored 5 different domains: the quality of reporting (10 items, maximum 11 points), the external validity (3 items, maximum 3 points), internal validity —bias (7 items, maximum 7 points), internal validity—confounding (selection bias; 6 items, maximum 6 points), and power (1 item with maximum 5 points). A summary of the set criteria (20 for human and 13 for mammalian) for quality assessments that were used is displayed in the Supplementary Table 1. The quality evaluation procedure was done independently by two reviewers (JS and AS), as previously advised (Wright et al., 1995; Harris et al., 2013). The level of agreement was assessed with Cohen's kappa analysis on all items of the checklist. Landis and Koch's benchmark for assessing agreement ranges from almost perfect (0.81–1.0), substantial (0.61–0.8), moderate (0.41–0.6), fair (0.21–0.4), slight (0.0–0.2), and poor (<0; Landis and Koch, 1977). Disagreements were resolved by consensus or by consulting a third reviewer.

## Results

### Study selection

**Figure 2:**
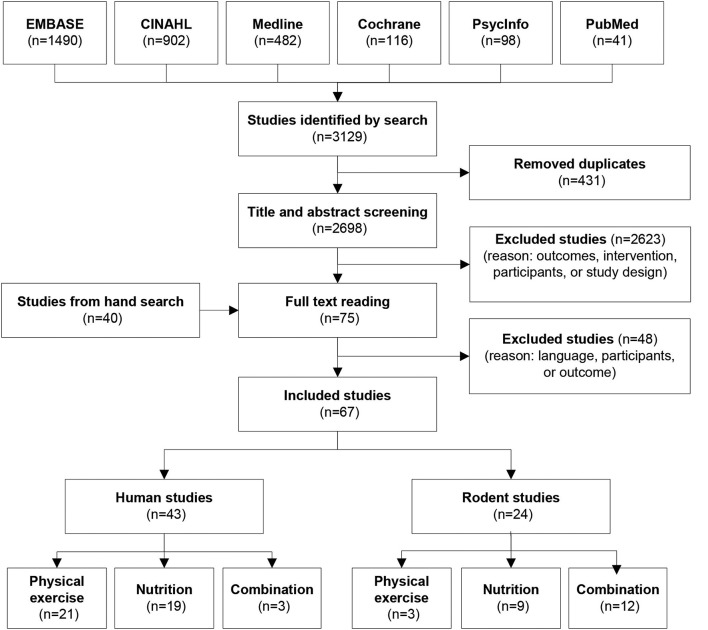
**Search and selection process**.

The database search resulted in a total number of 3129 studies. The selection process is illustrated in Figure 2. After the removal of duplicates (*n* = 431) and the screening process, 75 studies were left for full text reading. During full text reading, the cited human studies in the reference lists that perhaps would be relevant for the review were kept track leading to an additional 40 papers selected for full text reading. Finally, following full text reading, 67 articles were included in the systematic review. For the mammalian studies, the included studies were performed with rodents only.

### Study characteristics

#### Characteristics of the rodent studies

The total number of mice and rats in the 24 included studies was 1396 (median: 48 rodents per study, range 11–200). The studies examined C57BL/6 mice (Lee et al., 2002; Van der Borght et al., 2007; Kuhla et al., 2013), Wistar rats (Ito et al., 2009; Khabour et al., 2010, 2013; Jacotte-Simancas et al., 2013; Rachetti et al., 2013; Cechella et al., 2014a,b), Fischer 344 rats (Markowska, 1999), Long Evans rats (Young et al., 2007), Sprague-Dawley rats (Lee et al., 2000; Hansalik et al., 2006; Strasser et al., 2006; Wu et al., 2008; Chytrova et al., 2010; Gomez-Pinilla and Ying, 2010; Noble et al., 2014), F344xBN hybrid rats (Adams et al., 2008; Fitting et al., 2008; Carter et al., 2009), and BALB/cJ mice (Bhattacharya et al., 2015). At the beginning of the interventions, all rodents were disease free. The age of the rodents varied from a few months to a few years. The articles were studies that evaluated the effects of physical exercise (Van der Borght et al., 2007; Mustroph et al., 2012; Noble et al., 2014), of nutritional intervention (Markowska, 1999; Lee et al., 2000, 2002; Young et al., 2007; Adams et al., 2008; Fitting et al., 2008; Carter et al., 2009; Ito et al., 2009; Kuhla et al., 2013), or used a combination of physical exercise and nutritional intervention (Hansalik et al., 2006; Strasser et al., 2006; Wu et al., 2008; Chytrova et al., 2010; Gomez-Pinilla and Ying, 2010; Khabour et al., 2010; Jacotte-Simancas et al., 2013; Khabour et al., 2013; Rachetti et al., 2013; Cechella et al., 2014a,b; Bhattacharya et al., 2015). Physical exercise was always an aerobic type of exercise (running or swimming) performed five times per week for 20–60 min over a period of 2 weeks to 13 months, while nutritional interventions consisted of either dietary supplementation (taurin, niacin, amino acid, selenium, fatty acid, and epinephrine) or caloric restriction. Caloric restriction was included as a part of nutritional supplementation in the sense of nutritional depletion. A summary of measured outcomes in rodents is given in Table 2. The outcome categories that were assessed were behavior, neurogenesis, neurotrophins, synaptic proteins, cell signaling proteins, metabolic homeostasis proteins, and measures of oxidative stress.

**Table 2:** **Study outcomes measured in included rodent studies**.

**Category**	**Outcomes**	**Details**
Behavior	Behavioral tests	• Measurement of learning, memory, motor skill, and anxiety like behavior
Neurogenesis	Ki-67 staining	• Cellular marker for proliferation
	Doublecortin staining	• Marker for neurogenesis
	Immunohistochemistry	• Determination of phenotype of newly generated cells
		◦ GFAP: astrocyte protein
		◦ Neuronal nuclear marker
		◦ MAP2a: mature neuron-specific protein
Neurotrophins	BDNF-, NT-3-, trkB-, and trkC- mRNAs	• BDNF and NT-3: neurotrophins
		• TrkB and trkC: their high-affinity receptors
Synaptic proteins	NMDA receptor subunits:	• Glutamate receptor
	NR1, NR2A, and NR2B	• Important for synaptic plasticity and memory
	AMPA receptor subunits:	• Non-NMDA glutamate receptor
	GluR1 and GluR2	• Involved in plasticity and synaptic transmission
	Synaptophysin	• Involved in synaptic transmission
	STX-1 and STX-3	• Plasma membrane syntaxins
		• Present in synaptic membranes and in neuronal growth cones
	GAP-43	• Growth associated protein
	Synapsin	• Involvement in neurotransmitter release, axonal elongation, and maintenance of synaptic contacts
Cell signaling	CaMKII	• Signaling system
		• Important in learning and memory
	CREB staining	• Cellular transcription factor
		• Involvement in learning and memory
	Akt protein determination	• Involvement in cell signaling (cell proliferation)
Metabolic homeostasis	Glucocorticoids receptor, 11-beta-HSD1, ghrelin receptor, leptin receptor, p-AMPK, and SIRT1	• Molecular systems that play dual roles on metabolism and synaptic plasticity
Oxidative stress	Amount of oxidized proteins	• Measurement of oxidative stress

*11-beta-HSD, 11-beta-hydroxysteroid-dehydrogenase; AMPA, Alpha-amino-3-hydroxy-5-methyl-4-isoxazolepropionic acid; BDNF, Brain-derived neurotrophic factor; BrdU, Bromodeoxyuridine; CaMKII, Calmodulin-dependent protein kinase II; CREB, Cyclic adenosine monophosphate response element-binding protein; GAP-43, Growth associated protein 43; GFAP, Glial fibrillary acid protein; GluR 1/2, Glutamate receptor 1/2; MAP2a, Microtubule-associated protein 2a; mRNA, Messenger ribonucleic acid; NMDA, N-methyl-D-aspartate; NR1/2A/2B, N-methyl-D-aspartate receptor 1/2A/2B; NT-3, Neuriphin-3; p-AMPK, Phospho-adenosine monophosphate-activated protein kinase; SIRT1, Sirtuin 1; STX-1/-3, Syntaxin 1/3; trkB/C, Tropomyosin receptor kinase B/C*.

#### Characteristics of the human studies

The total number of participants in the 43 included human subject studies was 19,757. This number includes the participants from large supplementation studies such as the Women's Health Initiative WHI (*n* = 1420), the Physicians Health Study II PHSII (*n* = 5947), the Women's Health Study WHS (*n* = 6377), and the Age-Related Eye Disease Study AREDS (*n* = 2166). The number of participants decreased to 3847 (median: 58 participants per study, range 12–910) without the aforementioned studies. At the time of recruitment, all participants were healthy elderly living in a nursing home or at home and having a mean age around 65 years or higher. The studies evaluated the effects of physical exercise (Dustman et al., 1984; Blumenthal et al., 1991; Hill et al., 1993; Moul et al., 1995; Perrig-Chiello et al., 1998; Kramer et al., 1999; Baum et al., 2003; Bastone Ade and Jacob Filho, 2004; Colcombe et al., 2004, 2006; Ozkaya et al., 2005; Zlomanczuk et al., 2006; Cassilhas et al., 2007; Brown et al., 2009; Kamijo et al., 2009; Liu-Ambrose et al., 2010; Muscari et al., 2010; Erickson et al., 2011; Ruscheweyh et al., 2011; Voelcker-Rehage et al., 2011; Niemann et al., 2014), of nutritional intervention (Deijen et al., 1992; Smith A. et al., 1999; Smith A. P. et al., 1999; Cockle et al., 2000; Yaffe et al., 2004; Wolters et al., 2005; Kang et al., 2006; Grodstein et al., 2007; Malaguarnera et al., 2007; McMorris et al., 2007; McNeill et al., 2007; Summers et al., 2010; Presley et al., 2011; Macpherson et al., 2012; Rossom et al., 2012; Yasuno et al., 2012; Grodstein et al., 2013; Kelly et al., 2013; Szcześniak et al., 2014), or a combination of physical exercise and nutritional intervention (Cetin et al., 2010; Alves et al., 2013; van de Rest et al., 2014). Physical exercise in the human studies consisted of either strength or aerobic training that was usually done three times per week for 1 h over a period of 4–12 months. Nutritional intervention included (multi)vitamin supplementation, amino acids, nitrate enriched diets, creatine, fatty acid, or protein supplementation. Small nutritional supplementation studies were done over a period of several months, while large nutritional supplementation trials were done for several years. Many different outcomes have been measured in the human trials (Table 3). The focus of the majority of the articles was on cognitive tests. Briefly, cognitive tests were administered to the participants to assess general cognitive status, memory, executive function (EF), attention, intelligence, and sensorimotor performance. Measurements concerning the brain included brain volumes, brain activity, and metabolites concentrations in the brain. In addition, blood samples were used to measure serum BDNF, IGF-1, and neurotransmitter/hormone levels.

**Table 3:** **Study outcomes measured in included human studies**.

**Category**	**Outcome**	**Details**
Cognitive function	Cognitive tests	Tests for general cognitive functioning, memory, executive functions, intelligence, attention, and sensorimotor performance
Brain structure	Whole brain volume and regional brain volumes	MRI: voxel based morphometry
Brain activity	Electroencephalography: event-related potentials	During a cognitive task or a sensory stimulus (sensory evoked potential)
	Cerebral blood flow	Determined from MRI
	Apparent diffusion coefficients of white and gray matter	Acquired using an eight channel SENSE head coil
	Functional MRI: Cortical recruitment	Functional MRI during a cognitive task
Blood markers	Serum IGF-1, BDNF, dopamine, epinephrine and granulocyte colony-stimulating factor levels, and total antioxidant capacity	Blood samples (cephalic vein)
Metabolism	N-acetyl aspartate, creatine, choline, and myo-Inositol brain concentrations	^1^H magnetic resonance spectroscopy

*BDNF, Brain-derived neurotrophic factor; IGF-1, Insulin like growth factor 1; MRI, Magnetic resonance imaging*.

### Effects of physical exercise and nutritional intervention in rodents

The detailed results of the rodent combinatory studies are listed in Table 4. The detailed results of the rodent studies examining physical exercise or nutritional intervention are listed in the Supplementary Table 4. Ten studies evaluated the effects of physical exercise and nutritional supplementation on behavioral tests (Hansalik et al., 2006; Wu et al., 2008; Chytrova et al., 2010; Khabour et al., 2010, 2013; Jacotte-Simancas et al., 2013; Rachetti et al., 2013; Cechella et al., 2014a,b; Bhattacharya et al., 2015). Three out of these ten studies found additive effects of physical exercise and nutritional supplementation on Morris Water Maze (MWM; Wu et al., 2008) and object recognition task (Cechella et al., 2014a,b). Cechella et al. (2014a,b) found the benefits in 24 months old rats but not in 12 months old rats. However, the other seven studies found no additive beneficial effects on cognitive performance (Hansalik et al., 2006; Chytrova et al., 2010; Khabour et al., 2010, 2013; Jacotte-Simancas et al., 2013; Rachetti et al., 2013; Bhattacharya et al., 2015). Chytrova et al. (2010) and Rachetti et al. (2013) found that both physical exercise and nutritional supplementation resulted in improved learning or memory. However, Jacotte-Simancas et al. (2013) and Khabour et al. (2010, 2013) found that only exercised groups improved spatial learning and memory. On the other hand, Hansalik et al. (2006) found no improvements in the MWM for any of the intervention groups.

**Table 4:** **Included rodent studies combining physical exercise and nutritional intervention**.

**Study**	**Subjects**	**Intervention**	**Groups**	**Outcome measure**	**Results**
Bhattacharya et al., 2015	*N* = 91; male BALB/cJ mice Age: 10 weeks	Running wheel 1.49 mg of EGCG per g diet 3.34 mg of B-ALA per g diet	Exe, sed, B-ALA: exe or sed, EGCG: exe or sed, B-ALA + EGCG: exe or sed *N* = 11–12 per group	Fear conditioning (contextual and cued) BrdU staining	Exe increased duration of freezing (contextual) Exe approx. 4-fold greater duration of freezing behavior than sed (cued) No effect of diet or interaction between diet and exercise Exe increased total number of BrdU+ cells in the granule layer of the denate gyrus approx. 4 fold over sed No effect of diet or interaction between diet and exercise
Cechella et al., 2014a	Male wistar rats	Swim training: 20 min, 5×/week 1 ppm of diphenyl diselenide (selenium) 5 weeks	Exe (I), selenium (II), exe + selenium (III), adult control (IV), aged control (V) *N* = 4–6 per groups	ORT OLT	Short term memory: (I)+(II) improved compared to (V), (III) better than all other groups Long term memory: (II)+(III) better than control groups (II) better than aged control, (I) + (III) better than control groups
					
				CREB and Akt in hippocampus	pAkt/ Akt: same for (I)+(III)+(IV)+(V), (I) higher than control groups pCREB/ CREB: (I)+(II)+(III) better than aged control
Cechella et al., 2014b	*N* = 30; male wistar rats	Swim training: 20 min, 5×/week 1 ppm of diphenyl diselenide (selenium) 5 weeks	Exe (I), selenium (II), exe + selenium (III), adult control (IV), aged control (V) *N* = 4–6 per groups	ORT	Short term memory: (I)+(II)+(III) better than control groups, (III) shows the best results Long term memory: (I)+(II)+(III) better than aged control, (I) better than adult control
				OLT	(I)+(II)+(IIII) better than aged control
				CREB in hippocampus	pCREB/CREB: (I)+(II) better than control groups
Chytrova et al., 2010	*N* = 24; adult male Sprague-Dawley rats	Running wheel DHA enriched diet 12 days	RD + sed (I), DHA + sed (II), RD + exe (III), DHA + exe (IV) *N* = 6 per group	Synaptic proteins in hippocampus	NR2B: sig. increase for (II)+(III)+(IV), greatest effect for (IV) compared to (III) STX-3 and GAP-43: sig increase for (II)+(III)+(IV), greatest effect for (IV)
				MWM	Latency: (II)+(III)+(IV) decreased compared to (I)
Gomez-Pinilla and Ying, 2010	*N* = 24; male Sprague-Dawley rats	Running wheel DHA enriched diet 2 weeks	RD + sed (I), DHA + sed (II), RD + exe (III), DHA + exe (IV) *N* = 6 per group	Hip and Hyp dissection	Leptin: *Hyp*: increase for (II)+(III)+(IV), greatest for (IV), *Hip*: increase for (II) Ghrelin: *Hyp*: increase for (III), *Hip*: increase for (II)+(III) p-AMPK: *Hyp*: decrease for (II)+(III)+(IV), *Hip*: increase for (II)+(III)+(IV) SIRT: *Hyp*: increase for (II)+(III)+(IV), greatest for (IV), *Hip*: increase for (II)+(III)+(IV) Glucocorticoid receptor: *Hyp*: decrease for (II), increase for (III), *Hip*: increase for (II)+(III)+(IV) 11-beta-HSD1: *Hyp*: increase for (II), *Hip*: increase for (II)+(III)+(IV)
Hansalik et al., 2006	*N* = 200; male Sprague-Dawley rats	Running wheel Treadmill: 20 min, 5×/week CR 13 months	Baseline (age: 5 months) (I), exe (TM) (II), exe (RW) + CR (III), sed + CR (IV), sed1 (one rat, one cage) (V), sed4 (four rats, one cage) (VI)	MWM	Learning and short term memory: no effects comparing various intervention groups at age 10 and 18 months
Jacotte-Simancas et al., 2013	*N* = 62; male wistar rats Age: 2 months	Running wheel Epinephrine: 0.01 or 0.05 mg/kg	Sed (I), sed + 0.01 ep (II), sed + 0.05 ep (III), exe (IV), exe + 0.01 ep (V), exe + 0.05 ep (VI)	Barnes maze	Distance: (IV)+(V)+(VI) sig. shorter than (I)+(II)+(III) Latency: no sig. results
Khabour et al., 2010	Male wistar rats Age: 5 months	Voluntary exercise CR 6 weeks	Sed (I), CR (II), exe (III), exe + CR (IV) *N* = 10–13 per group	RAWM	Spatial learning and memory formation: (IV)+(III) enhanced compared to (I)+(II), no effect of CR
				Hippocampal BDNF	BDNF: (IV)+(III) sig. higher levels compared to (I)+(II), no effect of CR
Khabour et al., 2013	*N* = 92; young wistar male rats	Swimming: 60 min, 5×/week CR 6 weeks	Sed (I), CR (II), exe (III), exe + CR (IV) *N* = 15 per group	RAWM Hippocampal BDNF	Spatial learning and memory formation: (IV)+(III) enhanced learning/memory compared to (I)+(II), no effect of CR (IV)+(III) sig. higher levels compared to (I)+(II) no effect of CR
Rachetti et al., 2013	*N* = 45; adult wistar rats	Treadmill: 30 min, 5×/week (until age of 27 days) Fish oil capsules Length: prenatal to 10 months	Exe (I), exe + fish (II), control (III), control + fish (VI) *N* = 11–12 per group	Open field test	(VI) decrement in location during 2nd exposure compared to the other groups
				ORT	*Test session*: all groups explored sig. more the novel object compared to familiar object, *Re-test session*: (II) explored sig. more the novel object compared to familiar object
				Plus maze discriminative avoidance task	(I)+(II) discriminated the aversive from non-aversive arms and spent sig. less time in aversive arm
Strasser et al., 2006	Male Sprague-Dawley rats	Running wheel Treadmill: 20 min, 5×/week CR 13 months	Baseline (age: 5 months) (I), exe (TM) (II), exe (RW) + CR (III), sed + CR (IV), sed1 (one rat, one cage) (V), sed4 (four rats, one cage) (VI)	BDNF in parietotemporal cortex	Decrease for (V), increase for (VI), highest values for (VI)
Wu et al., 2008	*N* = 24; Sprague-Dawley rats	Running wheel DHA enriched diet 12 days	RD + sed (I), DHA + sed (II), RD + exe (III), DHA + exe (IV)	MWM BDNF, Synapsin I, CREB, Akt, CaMKII Oxidative proteins	Latency: (II)+(III)+(IV) shorter than (I), (IV) shorter than (II)+(III) (II)+(III) increased values and even more in (IV) (II)+(III) reduced oxidized protein and even more in (IV)

*The studies are reported by subjects, intervention, groups, outcome measure, and results. 11betaHSD1, 11-beta-hydroxysteroid-dehydrogenase type 1; B-ALA, Beta-alanine; BDNF, Brain-derived neurotrophic factor; BrdU, Bromodeoxyuridine; CaMKII, Calmodulin-dependent protein kinase II; CR, Calorie restriction; CREB, Cyclic adenosine monophosphate response element-binding protein; DHA, Docosahexaenoic acid; EGCG, Epigallocatechin gallate; Ep, Epinephrine; Exe, Exercise; GAP-43, Growth associated protein 43; Hip, Hippocampus; Hyp, Hypothalamus; MWM, Morris Water Maze; NR2B, N-methyl-D-aspartate Receptor 2B; OLT, Object Location Test; ORT, Object Recognition Test; p-AMPK, Phospho-adenosine monophosphate-activated protein kinase; pCREB, Phosphorylated cyclic adenosine monophosphate response element-binding protein; RAWM, Radial Arm Water Maze; RD, Restricted diet; RW, Running wheel; Sed, Sedentary; SIRT, Sirtuin; STX, Syntaxins; TM, Treadmill*.

Four articles assessed BDNF levels, three of them in the hippocampus (Wu et al., 2008; Khabour et al., 2010, 2013) and one in the cerebral parietotemporal cortex (Strasser et al., 2006). One study reported an additive effect (Wu et al., 2008), two studies found increased BDNF levels in the physical exercise group only (Khabour et al., 2010, 2013), and one study found no increased levels in any group. Moreover, two studies investigated the combinatory effect on synaptic proteins and both studies reported additive effects (Wu et al., 2008; Chytrova et al., 2010). In addition, three studies measured cyclic adenosine monophosphate response element-binding protein (CREB), Akt, or calmodulin-dependent protein kinase II (CaMKII) concentrations in the hippocampus (Wu et al., 2008; Cechella et al., 2014a,b). Wu et al. (2008) found that the combinatory intervention had higher cell signaling protein levels. However, Cechella et al. (2014a,b) measured increased CREB levels in the physical exercise or in the nutritional intervention group. Akt levels were only increased in the physical exercise group. Gomez-Pinilla and Ying found an inconsistent pattern of change in leptin and ghrelin receptor protein levels, phosphor-adenosine monophosphate-activated protein kinase, sirtuin 1, glucocorticoid receptor, and 11beta-hydroxysteroid dehydrogenase type 1 levels in the hypothalamus and in the hippocampus in rats (Gomez-Pinilla and Ying, 2010). At least, one study showed a positive effect of the combination group related to oxidative stress (decreased carbonyl levels; Wu et al., 2008).

### Effects of physical exercise and nutritional intervention in humans

The detailed results of the human studies that combined physical exercise with a nutritional intervention are listed in Table 5. The detailed results of the human studies examining physical exercise or nutritional intervention in isolation are listed in the Supplementary Table 5. Three studies evaluated the combined effects of physical exercise and nutritional supplements on cognitive functions (Cetin et al., 2010; Alves et al., 2013; van de Rest et al., 2014). None of these studies found additive effects of physical exercise and nutritional supplementation. Alves et al. investigated the effects of resistance training with creatine supplementation and found no change for any of the cognitive tests (Alves et al., 2013). Van de Rest et al. executed a similar protocol but with a protein drink (van de Rest et al., 2014). The results indicated that physical exercise in combination with protein and the group performing physical exercise only showed improvements in different cognitive domains. No interaction effect was found between the treatments. Cetin et al. looked at the effects of endurance exercise with vitamin E supplementation (Cetin et al., 2010). They found positive effects for the exercise group and no effect for the vitamin E supplementation for electroencephalography recordings. In addition, they found no changes in the antioxidant capacity for any intervention group (Cetin et al., 2010).

**Table 5:** **Included human studies combining physical exercise and nutritional intervention**.

**Study**	**Subjects**	**Intervention**	**Groups**	**Outcomes**	**Results**
Alves et al., 2013	*N* = 56; women Age range: 60–80 years	Strength exercise 3 sets for 7 exercise, 2 × week Creatine: 5 g/day 24 weeks	Creatine (I), exe (II), creatine + exe (III), non creatine + non exercise (IV) *N* = 14 per group	MMSE, Stroop test, TMT, Digit span test, delay recall test	No sig. diff. for any of the variables
Cetin et al., 2010	*N* = 57; sedentary Age range: 69.6–73.1 years	Aerobic exercise 90 min, 3 × week Vitamin E 6 months	Exe (I), vitamin (II), exe + vitamin (III), non exe + non vitamin (IV) *N* = 14–15 per group	EEG (auditory oddball paradigm) Plasma total antioxidant capacity	P3 amplitude: no diff. P3 latency: (I), (II), (III) shorter latency compared to Pre-treatment and (I)+(II) shorter latency compared to control No diff. to control group or within a group after 6 months
van de Rest et al., 2014	*N* = 127; frail and Pre-frail Mean age: 79 ± 8 years	Strength exercise 2 × week Protein shake: twice daily 24 weeks	Exe + protein (I), exe + placebo (II), non exe+ protein (III), non exe + placebo (IV) *N* = 62 (exe), 65 (non-exe)	Word learning test, Digit Span Task, TMT A&B, Stroop Color-Word Test, Verbal Fluency Test Finger Pre-cuing task Interaction effects exe-protein	(I) vs. (III): improvement: information processing speed (II) vs. (IV): improvement: attention, working memory Reaction time: improved over time in all groups No sig. interaction on any of the cognitive domains

*The studies are reported by subjects, intervention, groups, outcome measure, and results. EEG, Electroencephalography; Exe, Exercise; MMSE, Mini Mental Status Examination; TMT A/B, Trail Making Test A/B*.

### Quality evaluation

The agreement on study quality criteria between the two reviewers was substantial with an estimated Kappa value of 0.65 (95% confidence interval between 0.63 and 0.67). The percentage of agreement between the two raters was 85.15% for the human and rodent studies. The results of the physical exercise, nutrition, and combination intervention of human and rodent studies are summarized in the Supplementary Tables 2, 3.

#### Rodent studies

None of the 24 studies reached the maximum possible score of 13 points. The quality scores ranged from a minimum of 9 points to a maximum of 12. The mean quality score was 10.29 points (range: 9–12 points), the median value was 10.5 points and the mode was 11 points. The mean score for reporting was 5.04 points (maximum: 6 points; range: 4–6 points), for internal validity (bias) 4.08 points (maximum: 5 points; range: 4–5 points), for internal validity (bias) 0.58 (maximum: 1 points; range: 0–1 points), and for power 0.58 (maximum: 1 points; range: 0–1 points). The mean score for physical exercise studies was 11 points (maximum: 12 points; range: 10–12 points), for nutritional supplementation studies was 9.77 points (maximum: 11 points; range: 9–11 points), and for combination studies was 10.5 points (maximum: 12; range: 9–12 points).

#### Human studies

One study from the total 43 studies reached the maximum possible score of 21 points (Rossom et al., 2012). The average score was 14.12 points ranging from a minimum of 6 to a maximum of 21 points. The median value was 14 points and the mode was 15 points. The mean score for reporting was 7.21 points (maximum: 9 points; range: 3–9 points), for external validity 0.65 points (maximum: 2; range: 0–2 points), for internal validity (bias) 4.02 (maximum: 5 points; range: 2–5 points), for internal validity (confounding) 1.88 (maximum: 4 points; range: 0–4 points), and for power 0.35 (maximum: 1 points; range: 0–1 points). The mean score for physical exercise studies was 13.29 points (maximum: 20 points; range: 6–20 points), for nutritional supplementation studies was 14.68 points (maximum: 21 points; range: 10–21 points), and for combination studies was 16.33 points (maximum: 19; range: 15–19 points).

## Discussion

### Summary

The aim of this systematic review was to evaluate whether the combination of physical exercise and nutritional supplementation has greater benefits (additive effects) on brain structure and function than their separate administrations. Studies measured cognitive functioning with the help of behavioral tests and associated parameters including metabolic and structural neuronal changes on brain level. In human trials, the combination of physical exercise and nutritional supplementation did not lead to any additive effects. In rodents, four studies showed additive effects on different outcomes (Wu et al., 2008; Chytrova et al., 2010; Cechella et al., 2014a,b). Wu et al. showed additive effects on behavioral level, on BDNF level, on synaptic protein levels, and on oxidative stress using a combination of running exercise and docosahexaenoic acid (DHA) supplementation (Wu et al., 2008). Moreover, Chytrova et al. (2010) found additive effects on synaptic protein levels using a combination of running and DHA supplementation. Cechella et al. found additive effects on behavioral level using a combination of swim training and selenium (Cechella et al., 2014a,b).

The search strategy led to the identification of many articles with different brain function related outcomes. However, the studies that used a combination approach had a poor-moderate quality and were rather heterogenic which, in turn, led to difficulties comparing the results and performing a meta-analysis. The limited availability of high-quality prospective studies that used a combined approach warrants further targeted future research investigating the effects of combined approaches on the brain. Based on our findings we will discuss and suggest directions for future research related to combined interventions with physical exercise and nutritional supplements. Through this review it became apparent that isolated interventions of either physical exercise or nutrition were able to effect on brain in both mammals and humans. However, combinations of these components were not having an effect. It appears that many of the included studies have been using a “complementary” approach where the administration of physical exercise and nutritional components were not combined with the intention to cause an effect on similar mechanisms. No studies explicitly used an “additive” approach with the aim to enhance effects of the combined physical-exercise-&-nutrition-approach because both components shared similar mechanisms. For elucidating this seemingly contradiction we continue this discussion by considering the effects of studies applying either physical exercise or nutritional supplementations with the aim to effect on brain. Practices of various interventions are discussed together with the underlying mechanisms that theoretically would explain the effects of the used intervention components.

### Physical exercise interventions

Results from the three studies that looked at the effects of physical exercise on cognition in rodents showed better performances in learning and memory abilities. This finding is in agreement with existing literature reviews suggesting that physical exercise in rodents benefits cognitive functioning (van Praag, 2009; Gomez-Pinilla and Hillman, 2013). Interestingly, other studies showed that the physical exercise component, especially forced physical exercise, is able to activate neuronal brain metabolism (O'Callaghan et al., 2007; Kinni et al., 2011). Furthermore, providing opportunities for physical exercise is the critical element of environmental enrichment explaining the influence on neurogenesis (Kobilo et al., 2011). The mechanisms by which physical exercise improves cognition are, however, not yet fully understood. In the last couple of years, research seems to support the idea that physical exercise affects cellular and molecular systems associated with synaptic plasticity and energy metabolism (Gomez-Pinilla and Hillman, 2013). BDNF plays an essential role through the interaction with energy metabolism and growth factors (IGF-1) to influence downstream effectors mediating synaptic plasticity and neurogenesis, especially in the hippocampus (Gomez-Pinilla and Hillman, 2013). The hippocampus is an important area for learning and memory (Gomez-Pinilla and Hillman, 2013). In this systematic review, two studies found increased neurogenesis in the dentate gyrus of running mice (Van der Borght et al., 2007; Mustroph et al., 2012). In the hippocampus of running mice, Van der Borght et al. (2007) found increased phosphorylated CREB levels, a downstream effector of BDNF (Gómez-Pinilla, 2011).

In humans, aerobic exercise improved general cognitive functioning, EF, perceptual speed, and to some extent also memory. A meta-analytic review by Smith et al. found that aerobic exercise led to modest improvements in attention and processing speed, EF, and memory but did not affect working memory (Smith et al., 2010). In contrast, a systematic review by van Uffelen et al. found only weak evidence for better cognition after physical exercise; only 5 out of 15 studies showed significant improvements on some measures of cognition (van Uffelen et al., 2008). In addition, a meta-analysis by Colcombe et al. investigated the effects of fitness interventions on cognitive functions (Colcombe and Kramer, 2003). They showed that the largest benefits of physical exercise appear to be on EF. Hence, results from this systematic review and other reviews seem to support the idea that physical exercise has the most reliable effects on EF. Moreover, one might speculate that improvements in tests measuring general cognitive functioning might have been originated from improvements in subtests assessing EF. Although we do not know the sub-scores of the Mini Mental State Examination (MMSE), the study by Moul et al. using the Ross Information Processing Assessment to assess general cognitive functioning supports this hypothesis (Moul et al., 1995). The improved total score resulted from improvements in two attentional demanding tasks (organization and auditory processing). Differentiating the types of physical exercise, three studies found positive effects on memory examined following strength training. Two studies used strength training (Perrig-Chiello et al., 1998; Cassilhas et al., 2007) and one study combined strength training with endurance exercise (Zlomanczuk et al., 2006). Moreover, resistance training improved general cognitive functioning (Baum et al., 2003) and EF (Cassilhas et al., 2007; Liu-Ambrose et al., 2010). A review by Chang et al. on resistance exercise and cognition suggests that resistance exercise improves cognitive functions including information processing speed, attention, memory formation, and EF (Chang et al., 2012). Interestingly, participants who underwent coordination training improved in perceptual speed tasks (Voelcker-Rehage et al., 2011; Niemann et al., 2014).

The evaluation of neurogenesis and brain growth factors concentration is limited in human studies. In the studies included in our systematic review, the authors used magnetic resonance imaging techniques to assess brain volumes. Erickson et al. found that aerobic type of exercise was able to increase the hippocampus volume (Erickson et al., 2011). The results support the idea that physical exercise induces neurogenesis in the hippocampus. Furthermore, positive effects of aerobic type of exercise on brain volumes were found in the anterior cingulate cortex, the supplementary motor area, the right inferior frontal gyrus, the left superior temporal gyrus, and the anterior white matter (Colcombe et al., 2006; Ruscheweyh et al., 2011). The brain regions have been associated with critical cognitive processes (prefrontal cortex) and memory (temporal lobes; Colcombe et al., 2006). Moreover, the prefrontal cortex is considered to play an important role in EF (Funahashi, 2001) and has been shown to be susceptible to aging (Kamijo et al., 2009). In fact, Raz et al. argue that the brain regions that are late to mature (i.e., frontal regions) are also the most vulnerable to cognitive decline (Raz et al., 2005). In addition, cognitive aging seems to affect mainly tasks that require substantial mental effort and novel stimuli, such as EF, whereas semantic knowledge appears to be well preserved (Gunning-Dixon et al., 2009). On the other hand, strength training decreased whole brain volume (Liu-Ambrose et al., 2010). This finding seems paradoxical, since decreased brain volume is usually associated with impaired function (Carlson et al., 2008). However, Liu-Ambrose and colleagues concluded that this phenomenon needs to be further investigated (Liu-Ambrose et al., 2010). Interestingly, participants who underwent coordination training increased globus pallidus and caudate volumes (Voelcker-Rehage et al., 2011; Niemann et al., 2014). The two sub-regions of the basal ganglia are involved in prefrontal cognitive processes such as planning and working memory (Middleton and Strick, 1994). Since the dorsal part of basal ganglia is involved in motor learning (Niemann et al., 2014), it is not surprising that coordination training affected this area of the brain. This fact would also support the improvement in the perceptual speed task.

In an event-related potential study, latency of the P3 component has been attributed to information processing speed, attention, and working memory (Kügler et al., 1993). Ozkaya et al. found better early sensory processing for the strength but not for their endurance training group (Ozkaya et al., 2005). Kamijo et al. investigated the effects of one bout of aerobic exercise and found improved P3 latencies (Kamijo et al., 2009). Two studies used functional magnetic resonance imaging to evaluate brain activation during a flanker task (Colcombe et al., 2004; Voelcker-Rehage et al., 2011), and they showed greater activity in attentional control areas and reduced activity in the anterior cingulate cortex (ACC) that could be attributed to more efficient information processing (Voelcker-Rehage et al., 2011). Colcombe et al. argued that the successful completion of the incongruent flanker task requires activation of the frontal and parietal circuitry involved in spatial attention and a decreased activation of the ACC involved in response conflict (Colcombe et al., 2004).

With respect to blood markers, no increased serum BDNF or catecholamine levels were found after aerobic exercise. Erickson et al. found no significant changes in BDNF levels, but they found that changes in BDNF levels correlate with changes in hippocampal volume (Erickson et al., 2011). However, Ruscheweyh et al. also found no significant increase in BDNF levels after physical exercise (Ruscheweyh et al., 2011). The authors argued that the absence of increased BDNF levels in the blood could be due to two reasons: [1] levels could tailor off after approximately 1 month of training, [2] or cerebral BDNF levels might have been a better measure than blood levels to measure the impact of physical exercise on this parameter, although BDNF has been shown to pass the blood brain barrier. However, one study showed that resistance training for 6 months increased serum IGF-1 levels (Cassilhas et al., 2007), and this increase correlated with cognitive performance. The results support the hypothesis that IGF-1 plays an important role in cognition. Thus, more studies are needed to clarify the effect of aerobic exercise and resistance exercise on serum IGF-1 levels and on BDNF levels, respectively.

Overall, the results suggest that different types of physical exercise affect different cognitive domains through different mechanism. This is in line with previous research showing differing effects in the brain based on different exercise approaches. Where aerobic training increases activation in the sensorimotor network, coordination training leads to a higher activation of the visuospatial network (Voelcker-Rehage et al., 2011), and strength training has the potential to change the hemodynamic activity of brain regions associated with response inhibition processes (Liu-Ambrose et al., 2012). This is an indication that the types of physical training are likely to have task specific effects on the brain. Hence, combining aerobic exercise, strength training, and coordination training might be more beneficial for cognitive functioning than performing just one type of physical exercise. This kind of combined intervention was also suggested by Kramer et al. (1999). A recent study indeed demonstrated that a multicomponent simultaneous cognitive-physical training program was able to boost particularly EFs (including shifting attention and working memory) in healthy older adults compared to an exclusively physical multicomponent program (Eggenberger et al., 2015a), and that depending on the type of cognitive-physical training program applied differential training specific adaptations in brain function related walking parameters may be observed (Eggenberger et al., 2015b). However, it seems important that the principles of exercise training are consistently followed and accurately reported for physical exercise interventions (Ammann et al., 2014). Application of physical exercise principles (specificity, overload, progression, initial values, reversibility, and diminishing returns) ensures that the dose and type of physical exercise is planned to maximize the benefits for the recipients (Ammann et al., 2014). Such information was difficult to derive from the majority of studies included in this systematic review. It can be hypothesized that the lack of effect of physical exercise on the brain in some of the reports is partly due to not considering the quantity and quality of the exercise needed to trigger responses in the brain.

### Nutritional supplementation

In rodent studies, four out of five studies showed positive effects on learning, and only one study showed positive effects on memory using CR. However, these results have to be interpreted with caution. First of all, CR has also been shown to have negative effects on cognition. CR impairs memory assessed with object recognition (Carter et al., 2009) and increases anxiety like behavior, probably due to increased cortisol levels (Kuhla et al., 2013). Moreover, the study of Carter et al. evaluated whether the beneficial effects of CR are attributed to increased physical activity (Carter et al., 2009). They showed that CR rats had significantly higher activity levels than ad *libitum* (AL) fed rats. Moreover, the distance to reach the platform in the MWM task, which is not confounded by fitness, was the same in AL and CR rats. However, Kuhla et al. found improvements in spatial learning and working memory in CR rats that moved less than AL rats (Kuhla et al., 2013). Hence, the question whether CR has beneficial effects on cognition remains controversial. CR has often been used as an intervention because excess calorie intake might reduce synaptic plasticity through increased oxidative stress and subsequent cell damage (Gómez-Pinilla, 2008). In mice and rats, Lee et al. found that CR enhances neurogenesis by increasing survival of newly generated cells but not proliferation (Lee et al., 2000, 2002). This finding is interesting because physical exercise and CR appear to control different mechanisms; physical exercise increased newly generated cells in the hippocampus whereas CR promoted survival of cells in the hippocampus. The hypothesis that physical exercise is the strongest neurogenic stimulus is also supported by a review by van Praag on exercise and the brain (van Praag, 2009). If physical exercise and nutritional supplementation act differently on neurogenesis, their combined effects on neurogenesis could provoke additive results. However, a study by van Praag et al. showed that running increased both cell proliferation and survival in the hippocampus of mice (van Praag et al., 1999). Furthermore, the confounding effect of physical activity in CR mice or rats cannot be fully excluded (Lee et al., 2002). In addition to neurogenesis, Lee et al. showed increased BDNF (Lee et al., 2000, 2002) and NT-3 (Lee et al., 2002) levels in the hippocampus after CR (Lee et al., 2002). They argued that this might mediate the positive effects of CR on neurogenesis (Lee et al., 2002). These findings are in line with a review by Gomez-Pinilla on the effects of nutrients on brain function and a study by Duan et al. on CR (Duan et al., 2001; Gómez-Pinilla, 2008). Both studies suggested that CR increases BDNF levels and that this might mediate the effects on synaptic plasticity. Moreover, the authors illustrated that CR in rats was able to stabilize the decrease in key synaptic protein levels occurring with age. Again, these synaptic proteins are thought to be associated with synaptic plasticity in the hippocampus (Adams et al., 2008).

Studies that evaluated nutritional supplements showed no benefits for learning or memory using taurine or niacin (Young et al., 2007; Ito et al., 2009). For example, Young et al. showed that niacin supplementation worsened spatial learning ability, probably due to increased brain nicotinamide adenine dinucleotide and cyclic adenosine diphosphate ribose levels that facilitate long term depression and impair long term potentiation (Young et al., 2007). In addition, epinephrine supplementation did not lead to significant improvements in learning and memory (Jacotte-Simancas et al., 2013). On the other hand, DHA and diphenyl diselenide supplementation resulted in improved learning and memory (Wu et al., 2008; Chytrova et al., 2010; Gomez-Pinilla and Ying, 2010; Rachetti et al., 2013; Cechella et al., 2014a,b). The finding is in agreement with a review by Su that illustrated the positive effects of DHA on learning and memory performance in rodents (Su, 2010). Results from this systematic review suggest that the effects of the diphenyl diselenide supplementation on learning and memory involve CREB phosphorylation without altering the levels of Akt (Cechella et al., 2014b).

In humans, vitamin and multivitamin supplementation did not seem to positively affect scores of cognitive tests. No evidence was found for EF, processing speed, attention, or intelligence. A systematic review and meta-analysis by Grima et al. on the effects of multivitamin supplementation on cognitive performance revealed minimal benefits after vitamin supplementation (Grima et al., 2012). They showed that only immediate free recall memory seemed to profit from vitamin supplementation but not the other cognitive domains. On the other hand, cross sectional studies show associations between vitamin status and cognitive functioning. For example, a recent systematic review showed that low vitamin D status is associated with lower outcomes in cognitive tests (van der Schaft et al., 2013). In addition, Cockle et al. list many other studies that showed associations between vitamin status and cognitive functioning, for example vitamin B12 and memory or folate and spatial copying ability (Cockle et al., 2000). Smith et al. found no improvements in cognitive functions after multivitamin supplementation. However, a subgroup analysis revealed that individuals with low baseline levels of vitamin C improved in cognition after the supplementation (Smith A. P. et al., 1999). We think that the discrepancy between cross sectional and interventional studies arises because studies in our systematic review investigated the possible causal effects of supplementation in healthy individuals without known vitamin deficiencies.

The other supplementation studies showed different effects on cognitive functioning in humans. L-carnitine or anserine plus carnosine improved MMSE scores. It is noteworthy that improvements were only seen in very old people. For example, Malaguarnera et al. (2007) investigated the effects of L-carnitine on centenarians and Szcześniak et al. (2014) found improved MMSE scores after anserine and carnosine supplementation only in people aged 81–94 but not in those aged 65–80 (Malaguarnera et al., 2007; Chytrova et al., 2010). In addition, participants from the study by Malaguarnera et al. (2007) had very low baseline scores of the MMSE, averaging 16.5 points. Furthermore, fish oil together with lycopene and gingko biloba improved general cognitive functioning, memory, and processing speed and attention (Yasuno et al., 2012). Other studies that investigated the effects of n-3 polyunsaturated fatty acid on cognitive functions found no positive effects on cognition (Rogers et al., 2008; Quinn et al., 2010). McMorris et al. found that creatine supplementation improved memory scores, but the study quality was rather low (McMorris et al., 2007). A higher ranked study (15 points) combined creatine with resistance exercise and reported no improvement in memory, EF, or MMSE in the creatine group (Alves et al., 2013). In addition, NO_3_ supplementation showed no beneficial effects on cognition (Kelly et al., 2013). The inconsistent results of this systematic review limit the strength of the evidence that supports the intake of supplements on cognition.

Our search strategy detected no studies that evaluated the effects of nutritional supplementation on brain volumes or neurotrophin blood levels in humans due to the fact that the proposed mechanisms of supplements, especially of vitamin supplementation, usually involve antioxidant properties. Antioxidant foods have been claimed to favor cognition because of the susceptibility of the brain to oxidative damage (Gómez-Pinilla, 2008). However, in our systematic review few antioxidant supplements had positive effects on cognition. Reasons could be that participants were too healthy (Cockle et al., 2000; Kelly et al., 2013), In addition, antioxidant supplementation might protect against the deleterious effects of diets rich in saturated fats and sugars which have been shown to increase oxidative stress and decrease hippocampal BDNF levels (Gómez-Pinilla, 2011). Overall, results from vitamin studies seem to support the idea that vitamin supplementation is beneficial for cognition only in participants with low baseline vitamin status.

Two studies hypothesized that nitrate would be converted to nitric oxide that results in a vasodilation and consequently increases blood flow to the brain (Presley et al., 2011; Kelly et al., 2013). However, Kelly et al. found no positive effect on any of the measured outcomes (cognitive tests, apparent diffusion coefficients, and brain metabolite concentrations; Kelly et al., 2013). Furthermore, Presley et al. found no differences in global perfusion (Presley et al., 2011). Both studies used very short intervention periods of two to two-and-a-half days. Hence, studies with longer NO_3_ supplementation periods seem necessary to evaluate long term effects on brain perfusion and cognition (Kelly et al., 2013).

The search strategy did not yield studies that investigated the effects of CR in elderly humans. However, hand searching yielded a study that showed beneficial effects of CR (30% reduction) on memory performance in healthy elderly (Witte et al., 2009). Higher synaptic plasticity and stimulation of neurofacilitatory pathways might be due to improved insulin sensitivity and reduced inflammatory activity.

### Combination of physical exercise and nutritional supplementation

In rodent studies, DHA or diphenyl diselenide in combination with physical exercise evoked additive effects (Wu et al., 2008; Chytrova et al., 2010; Cechella et al., 2014a,b). DHA is a dietary omega-3 fatty acid and has the potential to affect synaptic plasticity and cognition (Gómez-Pinilla, 2008, 2011). A review on brain foods described why DHA is important for cognition and brain heath: DHA constitutes more than 30% of phospholipids of plasma membranes of neurons, and thus plays a crucial role for synaptic function (Gómez-Pinilla, 2008). More importantly, DHA can affect molecules such as BDNF and IGF-1 which in turn can activate signaling systems such as mitogen-activated protein kinase, CaMKII, and phosphoinositide 3-kinase/Akt/mammalian target of rapamycin (Gómez-Pinilla, 2008). Therefore, DHA seems to have the potential to facilitate synaptic transmission, to modulate synaptic plasticity and cognitive function, and to support long term potentiation which is associated with learning and memory (Gómez-Pinilla, 2008). Interestingly, the effects of physical exercise seem to depend on similar mechanisms involving BDNF mediated synaptic plasticity and energy homeostasis (Gómez-Pinilla, 2011). Thus, DHA supplementation could complement the actions of physical exercise resulting in an effective strategy to counteract cognitive decline (Gómez-Pinilla, 2011). In both studies, the combination had greater effects on hippocampal BDNF levels, synaptic protein and signaling molecules levels, on proteins involved in metabolic homeostasis, and on oxidative stress (Wu et al., 2008; Chytrova et al., 2010). However, the positive effects were only seen in two studies (Wu et al., 2008; Chytrova et al., 2010) which used an identical design: 12 days of 1.25% DHA supplementation with or without free access to a running wheel on 24 Sprague-Dawley rats. Both studies using diphenyl diselenide and swimming expected increased CREB level as mediator for improved memory (Cechella et al., 2014a,b). As the CREB levels did not change, the question about the underlying neurobiological mechanism remains to be elucidated.

In humans, none of the three studies that combined physical exercise and nutritional supplementation showed additive effects (Cetin et al., 2010; Alves et al., 2013; van de Rest et al., 2014). The reason for no additive effects might be that the combination of intervention components was not explicitly selected based on a shared mechanism and, therefore, evoked complementary effects at best. This means that the chosen single components (physical exercise or nutritional intervention) of the combined administration act not on the same neurobiological cascade to produce additive effects.

To evoke possible additive effects, three items seemingly should be taken into account: [1] training principles to ensure quality and quantity of the exercise component, [2] dose and duration of diet or nutritional supplementation, and [3] the selected nutritional component(s) and physical exercise should act on the same neurobiological cascade. The results can be interpreted in the sense that so far there seems to be a mismatch in many studies between the exercise program offered and the nutritional supplements given. It seems reasonable to assume that the nutritional supplements should be selected based on the theoretical effect they have on the brain; e.g., they preferably should share similar mechanisms with exercise (Gómez-Pinilla, 2011) and, thus, theoretically have the potential to complement the action of exercise. Possible additive effects might be achieved, if both components (physical exercise and nutrition) act complementary on the same molecular mechanisms. Other influencing factors are the genetic component and the living environment that are very individual in humans, but more or less identical in rodents. In humans, the individual genetic variability influences individual response to nutritional intervention (Dauncey, 2015).

### Strengths and limitations

#### Review

The standards of reporting animal experiments lag behind those of human RCTs (Muhlhausler et al., 2013), which is a potential concern for bias. Furthermore, publication bias related to overstatement of efficacy may negatively affect the interpretation of animal studies (Sena et al., 2010). A further limitation relates to the focus on older animals and humans. The precise correlation between the age of rodents and humans is subject of debate implying, when age is an important factor, differences between animals and humans should be taken into consideration (Sengupta, 2013).

For the human trials, the majority of included studies resulted from the reference list search. Hence, we cannot guarantee that all studies examining nutritional supplementation and physical exercise on cognition in healthy elderly are included in this systematic review. Moreover, we performed no gray literature search, and thus cannot guarantee that there was no publication bias. Another limitation is that the included studies are very heterogeneous regarding included participants, interventional design, and outcomes. This heterogeneity hindered a meta-analytical approach which would have been a more objective way to quantify the results.

#### Individual studies

Generally, the included studies in this systematic review were of good quality. In the quality evaluation of rodent studies, the question assessing power had a low average score because it was often not possible to be determined (the results were displayed in graphs and not tabulated). Question 25 addressing confounding averaged only 0.58 points due to insufficient information regarding the number of rodents that were used for the analysis. The small differences in scores between studies might be explained through the very similar study designs. However, in human studies quality scores varied much more, probably due to heterogeneous study designs. Moreover, it is important to keep in mind that quality scores rely upon the quality of reporting rather than the quality of the actual study conduct (Harris et al., 2013).

Furthermore, the number of cognitive tests used in human studies is huge, and almost every study used different tests. Moreover, it was common that some authors used the same specific test but evaluated different cognitive domains. In other words, no standardized way is present to evaluate cognitive functioning. In addition, it can be argued that the applied tests were not always appropriate causing a suspected misfit between the targets of the intervention and the used (un)specific outcome tests. For example, many authors used the MMSE to evaluate general cognitive functioning. However, the MMSE is a diagnostic tool not designed to measure change or improvement and might, therefore, suffer from ceiling effects (Summers et al., 2010). In animals, the MWM was often used to evaluate learning and memory. However, Fitting et al. suggested that the improvements in latency after CR were due to preservation of motor function and not due to cognition (Fitting et al., 2008). In addition, Jacotte-Simancas et al. argued that exercised animals might perform better than sedentary animals in the MWM because they cope better with the physical effort and stress generated by the task (Jacotte-Simancas et al., 2013). Hence, motor fitness could be a confounding factor for cognitive performance evaluation in the MWM. An additional possible limitation of the studies with rodents relates to the gender distribution of the investigated animals. The fast majority of the animal studies identified through the systematic literature search were carried out on male animals only. From human studies we know that some brain related impairments affect women more than men; e.g., sex disparity in stroke prevalence persists with women being more affected then men (Towfighi et al., 2011). It seems important that future studies test interventions in both sexes.

### Conclusion

In healthy elderly humans, no additive effects were identified for nutritional supplementation and physical exercise. In rodents, DHA and physical exercise or selenium and physical exercise resulted in additive effects on learning and on neurobiological measures. The main interventions that resulted in improved cognition or associated parameters were aerobic type of exercise, strength training, coordination training, CR, and DHA supplementation. It can, thus, be speculated that a combination of these interventions might provide better cognitive outcomes than just their sole administration. More research is needed examining the possible additive effects of physical exercise and nutritional intervention in humans. This systematic review reveals that applications of targeted exercise in combination with nutritional supplements with the aim to effect on the brain are still at a fledgling stage. There are, however, interesting first results in rodent studies that encourage further work in this field and which hold promise for utilizing the combined exercise-nutrition approach as a therapeutic tool.

### Future direction

A central element of successful cognitive rehabilitation for older adults should be the design of interventions that either re-activate disused or damaged brain regions, or that compensates for decline in parts of the brain through the activation of compensatory neural reserves (Hogan, 2005). Based on the results of the systematic review, we would design further combinatory studies as follows: Based on the findings of this review the nutritional supplements should be selected such that they share similar mechanisms with exercise and, thus, theoretically have the potential to support the action of exercise. Physical exercise would base on a combination of aerobic and strength exercise that also includes a cognitive component. Considering the cognitive part, previous research suggests a focus on executive functioning processes including enriched environments that provide physical activities with decision-making opportunities because these are believed to be able to facilitate the development of both motor performance and brain functions (Yan and Zhou, 2009). The use of virtual reality environments for virtual augmented exercise has recently been proposed as having the potential to increase exercise behavior in older adults in combination with the potential to influence cognitive abilities (de Bruin et al., 2010). At present there is evidence that specific types of video games are able altering brain structure (Shams et al., 2015) and function (Eggenberger et al., 2016) and, when added to a multicomponent exercise program, improve certain aspects of cognitive functioning (Eggenberger et al., 2015a,b). Future research should develop, implement, and evaluate for example virtual reality based training scenarios that allows the combination of aerobic and strength exercises together with cognition. Moreover, video games allow the implementation of FITT (Frequency, Intensity, Type, and Time) training principles to ensure that the dose and type of physical exercise is planned to maximize the benefits for the recipients. For nutrition, a diet including omega-3 fatty acid, is assumed to have the potential to affect synaptic plasticity and cognition. A previous study performed in humans showed beneficial effects on cognitive functioning and memory (Witte et al., 2014). Furthermore, one study investigated the effects of CR in healthy elderly humans (Witte et al., 2009). In rodents, CR showed positive effects on brain function, but CR studies should be controlled for the confounding factor of increased physical activity.

In future studies, authors should agree upon a standardized set of tests in order to compare the results between studies, since there is a myriad of tasks that have been proposed to evaluate cognitive functioning. However, physical activity and nutrition are closely linked together, and positive effects of physical exercise might be confounded by better nutrition. Controlling for this factor appears necessary, if one wants to evaluate the additive effects of physical exercise and nutrition on cognition.

## Author contributions

AS, KB, and JS developed the research question under the lead of PW and ED. The concept and design part was established by AS, KB, and JS while PW and ED acted as methodological councils. AS, KB, and JS did articles acquisition as well as analysis and interpretation of the articles which was edited and improved by PW and ED. AS and JS produced an early version of the manuscript. KB, PW, and ED substantially revised the manuscript to bring it to its current version. All authors have read and approved the final manuscript.

### Conflict of interest statement

The authors declare that the research was conducted in the absence of any commercial or financial relationships that could be construed as a potential conflict of interest.
