# Supplementary material for: Effects of Physical Exercise Combined with Nutritional Supplements on Aging Brain Related Structures and Functions: A Systematic Review
Source: Front Aging Neurosci. 2016 Jul 6;8:161. doi: 10.3389/fnagi.2016.00161 (PMC4933713; doi:10.3389/fnagi.2016.00161)
Supplement: Supplementary file 1 [file Table1.pdf]

Supplementary Material

Effects of physical exercise combined with nutritional supplements on aging brain related structures and functions: A systematic review

Alexandra Schättin[*+], Kilian Baur[2+], Jan Stutz[1], Peter Wolf[2], Eling D. de Bruin[1]

[1] Department of Health Sciences and Technology, Institute of Human Movement Sciences and Sport, ETH Zürich, HIT J 32, Wolfgang-Pauli-Str. 27, 8093 Zurich, Switzerland

[2] Department of Health Sciences and Technology, Sensory-Motor Systems Lab, ETH Zürich, TAN E 4, Tannenstrasse 1, 8092 Zurich, Switzerland

[+] shared first author

*** Correspondence:** Alexandra Schättin: schaetta@hest.ethz.ch

Supplementary Table

Supplementary table 1. Included quality criteria for the human (● ○) and mammalian (○) studies. The questions are chosen from the Downs & Black checklist for randomized and non-randomized studies of health care interventions. The numbers in brackets correspond to the official questionnaire numbers of the Downs & Black checklist. Each question can have 0 (no or unable to determine) or 1 (yes) points except question 5 of reporting can have 0 (no), 1 (partially), or 2 (yes) points.

Reporting	
o	(1) Is the hypothesis/aim/objective of the study clearly described?
o	(2)Are the main outcomes to be measured clearly described in the introduction or the methods section?
•	(3) Are the characteristics of the subjects included in the study clearly described?
o	(4) Are the interventions of interest clearly described?
•	(5) Are the distributions of principal confounders in each group of subjects to be compared clearly described?
o	(6) Are the main findings of the study clearly described?
o	(7) Does the study provide estimates of the random variability in the data for the main outcomes?
o	(10) Have actual probability values been reported (e.g. 0.0035 rather than <0.05) for the main outcomes except where the probability value is less than 0.001?
External validity	
•	(11) Were the subjects asked to participate in the study representative of the entire population from which they were recruited
•	(12) Were those subjects who were prepared to participate representative of the entire population from which they were recruited?
Internal validity – bias	
o	(15) Was an attempt made to blind those measuring the main outcome of the intervention?
o	(16) If any of the results of the study were based on “data dredging” was this made clear?
o	(18) Were the statistical tests used to assess the main outcomes appropriate?
o	(19) Was compliance with the intervention/s reliable?

- (20) Were the main outcome measures used accurate (valid and reliable)?

Internal validity – confounding

- (21) Were the patients in different intervention groups (trails and cohort studies) or were the cases and controls (case-control studies) recruited from the same population?
- (22) Were study subjects in different intervention groups (trails and cohort studies) or were the cases and controls (case-control studies) recruited over the same period of time?
- (23) Were study subjects randomized to intervention groups?
- (25) Was there adequate adjustment for confounding analyses from which the main findings were drawn?

Power

- (27) Did the study have sufficient power to detect a clinically important effect where the probability value for a difference being due to chance is less than 5 %?
