# Supplementary material for: Effects of Physical Exercise Combined with Nutritional Supplements on Aging Brain Related Structures and Functions: A Systematic Review
Source: Front Aging Neurosci. 2016 Jul 6;8:161. doi: 10.3389/fnagi.2016.00161 (PMC4933713; doi:10.3389/fnagi.2016.00161)
Supplement: Supplementary file 2 [file Table2.pdf]

Supplementary Material

Effects of physical exercise combined with nutritional supplements on aging brain related structures and functions: A systematic review

Alexandra Schättin[*+], Kilian Baur[2+], Jan Stutz[1], Peter Wolf[2], Eling D. de Bruin[1]

[1] Department of Health Sciences and Technology, Institute of Human Movement Sciences and Sport, ETH Zürich, HIT J 32, Wolfgang-Pauli-Str. 27, 8093 Zurich, Switzerland

[2] Department of Health Sciences and Technology, Sensory-Motor Systems Lab, ETH Zürich, TAN E 4, Tannenstrasse 1, 8092 Zurich, Switzerland

[+] shared first author

*** Correspondence:** Alexandra Schättin: schaetta@hest.ethz.ch

Supplementary Table

Supplementary table 2. Results of the quality evaluation of included human studies. The questions are chosen from the Downs & Black checklist for randomized and non-randomized studies of health care interventions. The question number corresponds to the official questionnaire numbers of the Downs & Black checklist. Each question can have 0 (no or unable to determine) or 1 (yes) points except question 5 of reporting can have 0 (no), 1 (partially), or 2 (yes) points.

Question number	1	2	3	4	5	6	7	10	SUM	11	12	SUM	15	16	18	19	20	SUM	21	22	23	25	SUM	27	Total
**Physical exercise**																									
Bastone and Filho 2004	1	1	1	1	1	1	1	1	8	1	1	2	0	1	1	0	1	3	1	0	0	0	1	0	14
Baum et al. 2003	1	1	1	1	1	1	1	1	8	1	1	2	1	1	1	0	1	4	1	0	1	1	3	1	18
Blumenthal et al. 1991	1	1	0	1	0	1	1	0	5	0	1	1	0	1	1	1	1	4	1	0	1	1	3	0	13
Brown et al. 2009	1	1	1	1	2	1	1	1	9	1	1	2	0	1	1	1	1	4	1	0	1	0	2	1	18
Cassilhas et al. 2007	1	1	1	1	1	1	1	1	8	0	0	0	0	1	1	1	1	4	0	0	1	1	2	1	15

Colcombe et al. 2004	1	1	0	1	1	0	1	0	5	0	0	0	0	1	1	0	1	3	0	0	1	0	1	0	9
Colcombe et al. 2006	1	1	0	1	1	1	1	0	6	0	0	0	1	1	1	1	1	5	0	0	1	0	1	0	12
Dustman et al. 1984	1	1	0	1	1	1	1	0	6	0	0	0	0	1	1	0	1	3	0	0	1	0	1	0	10
Erickson et al. 2011	1	1	1	1	1	1	1	0	7	0	1	1	1	1	1	1	1	5	1	0	1	0	2	0	15
Hill et al. 1993	1	1	1	1	0	1	1	0	6	0	0	0	0	1	1	0	1	3	0	0	0	0	0	0	9
Kamijo et al. 2009	1	1	0	1	2	1	1	1	8	0	0	0	0	1	1	1	1	4	0	0	0	0	0	0	12
Kramer et al. 1999	1	1	0	0	0	1	0	0	3	0	0	0	0	1	0	0	1	2	0	0	1	0	1	0	6
Liu-Ambrose et al. 2010	1	1	1	1	2	1	1	0	8	0	0	0	1	1	1	0	1	4	1	1	1	1	4	0	16
Moul et al. 1995	1	1	1	1	1	1	1	0	7	0	0	0	0	1	1	0	1	3	0	0	1	0	1	0	11
Muscari et al. 2010	1	1	1	1	2	1	1	1	9	1	1	2	1	1	1	1	1	5	1	1	1	1	4	0	20
Niemann et al. 2014	1	1	1	1	2	1	1	1	9	0	1	1	1	1	1	1	1	5	1	0	1	0	2	0	17
Ozkaya et al. 2005	1	1	1	1	2	1	1	1	9	0	1	1	0	1	1	0	1	3	0	0	1	0	1	1	15
Perrig-Chiello et al. 1998	1	1	0	1	0	1	1	1	6	1	1	2	0	1	1	0	0	2	1	0	1	0	2	0	12
Ruscheweyh et al. 2011	1	1	1	1	2	1	1	0	8	0	0	0	1	1	1	1	1	5	0	0	1	0	1	1	15
Voelcker-Rehage and Staudinger 2011	1	1	1	1	2	1	1	1	9	0	0	0	0	1	1	0	1	3	1	0	1	0	2	0	14
Zlomanczuk et al. 2006	1	1	0	0	0	1	1	0	4	0	0	0	0	1	1	0	1	3	1	0	0	0	1	0	8
**Mean**									**7.05**			**0.67**						**3.67**					**1.67**	**0.24**	**13.29**
**Min**									**3**			**0**						**2**					**0**	**0**	**6**
**Max**									**9**			**2**						**5**					**4**	**1**	**20**
**Nutrition**																									
Cockle et al. 2000	1	1	0	1	1	1	1	0	6	0	0	0	1	1	1	1	1	5	1	0	1	0	2	0	13
Deijen et al. 1992	1	1	1	1	1	1	1	0	7	0	1	1	1	1	1	1	1	5	0	0	1	0	1	0	14
Gordstein et al. 2013	0	1	1	0	2	1	1	0	6	1	1	2	1	1	1	1	1	5	1	1	1	0	3	1	17
Grodstein et al. 2007	0	1	0	1	2	1	1	1	7	0	0	0	0	1	1	0	1	3	0	1	1	1	3	1	14
Kang et al. 2006	1	1	1	1	2	1	1	1	9	0	0	0	1	1	1	1	1	5	0	0	1	0	1	1	16
Kelly et al. 2012	1	1	0	1	0	1	1	0	5	0	0	0	1	1	1	1	1	5	0	0	1	0	1	0	11
Macpherson et al. 2012	1	1	1	1	1	1	1	1	8	1	1	2	1	1	1	1	1	5	1	1	1	0	3	0	18
Malaguarnera et al. 2007	1	1	1	1	2	1	1	0	8	0	1	1	1	1	1	1	1	5	1	1	1	1	4	1	19
McMorris et al. 2007	1	1	0	1	0	1	1	0	5	0	0	0	1	1	1	0	1	4	0	0	0	0	0	1	10
McNeill et al. 2007	1	1	0	1	2	1	1	0	7	1	1	2	1	1	1	1	1	5	1	1	1	1	4	0	18
Presley et al. 2011	1	1	1	1	0	1	1	1	7	0	0	0	0	1	1	1	1	4	0	0	1	0	1	0	12
Rossom et al. 2012	1	1	1	1	2	1	1	1	9	1	1	2	1	1	1	1	1	5	1	1	1	1	4	1	21
Smith et al. 1999	1	1	1	1	1	1	1	0	7	0	0	0	1	1	1	0	1	4	0	0	1	0	1	1	13

Smith et al. 1999	1	1	1	1	1	1	1	0	7	0	0	0	1	1	1	0	1	4	0	0	1	0	1	1	13
Summers et al. 2010	1	1	1	1	1	1	1	1	8	0	0	0	1	1	1	0	1	4	1	0	1	0	2	0	14
Szczesniak et al. 2014	1	1	1	1	1	1	1	1	8	1	1	2	1	1	1	0	1	4	1	0	1	0	2	0	16
Wolters et al. 2005	1	1	1	1	2	1	1	1	9	0	0	0	1	1	1	1	1	5	1	0	1	0	2	0	16
Yaffe et al. 2004	1	1	0	1	0	1	1	0	5	0	0	0	0	1	1	0	0	2	1	1	1	1	4	0	11
Yasuno et al. 2012	1	1	1	1	2	1	1	1	9	0	0	0	0	1	1	1	0	3	1	0	0	0	1	0	13
**Mean**									**7.21**			**0.63**						**4.32**					**2.11**	**0.42**	**14.68**
**Min**									**5**			**0**						**2**					**0**	**0**	**10**
**Max**									**9**			**2**						**5**					**4**	**1**	**21**
**Combination**																									
Alves et al. 2013	1	1	1	1	1	1	1	1	8	0	0	0	1	1	1	1	1	5	0	0	1	0	1	1	15
Cetin et al. 2010	1	1	1	1	1	1	1	1	8	0	1	1	0	1	1	1	1	4	1	0	1	0	2	0	15
van de Rest et al. 2013	1	1	1	1	2	1	1	1	9	0	1	1	1	1	1	1	1	5	1	1	1	0	3	1	19
**Mean**									**8.33**			**0.67**						**4.67**					**2.00**	**0.67**	**16.33**
**Min**									**8**			**0**						**4**					**1**	**0**	**15**
**Max**									**9**			**1**						**5**					**3**	**1**	**19**
**Mean total**									**7.21**			**0.65**						**4.02**					**1.88**	**0.35**	**14.12**
**Min total**									**3**			**0**						**2**					**0**	**0**	**6**
**Max total**									**9**			**2**						**5**					**4**	**1**	**21**
**Median total**									**8**			**0**						**4**					**2**	**0**	**14**
**Mode total**									**8**			**0**						**5**					**1**	**0**	**15**
