# Supplementary material for: Effects of Physical Exercise Combined with Nutritional Supplements on Aging Brain Related Structures and Functions: A Systematic Review
Source: Front Aging Neurosci. 2016 Jul 6;8:161. doi: 10.3389/fnagi.2016.00161 (PMC4933713; doi:10.3389/fnagi.2016.00161)
Supplement: Supplementary file 4 [file Table4.pdf]

Supplementary Material

Effects of physical exercise combined with nutritional supplements on aging brain related structures and functions: A systematic review

Alexandra Schättin[*+], Kilian Baur[2+], Jan Stutz[1], Peter Wolf[2], Eling D. de Bruin[1]

[1] Department of Health Sciences and Technology, Institute of Human Movement Sciences and Sport, ETH Zürich, HIT J 32, Wolfgang-Pauli-Str. 27, 8093 Zurich, Switzerland

[2] Department of Health Sciences and Technology, Sensory-Motor Systems Lab, ETH Zürich, TAN E 4, Tannenstrasse 1, 8092 Zurich, Switzerland

[+] shared first author

*** Correspondence:** Alexandra Schättin: schaetta@hest.ethz.ch

Supplementary Table

Supplementary table 4. Included rodent studies for physical exercise or nutritional intervention. The studies are reported by subjects, intervention, groups, outcome measure, and results.

Study	Subjects	Intervention	Groups	Outcome measure	Results
**Physical exercise**					
Mustropf et al, 2012	- N=32; male mice<br>- Age: 7 weeks	- Running wheel<br>- Enriched environment<br>- 32 days	- EE (I), exe (II), EE + exe (III), control (IV)<br>- N= 8 per group	- Hippocampal neurogenesis<br><br>- MWM	- (II) increase in total number of BrdU<br>- (I)+(II) increase of new neurons in dentate gyrus<br>- (II): steepest learning curve in MWM<br>- (II): steepest learning curve in MWM

Noble et al, 2014 (Experiment 1)	- N= 29 ; male Sprague-Dawley rats<br>- Age: 6 months	- Running wheel<br>- Treadmill exercise: 45min, 5×/ week<br>- 5 weeks	- Exe (RW) (I), exe (TM) (II), sed (III)<br>- N = 9-10	- Two-way active avoidance task	- (I)+(II): sig. effect on latency, number of escapes, avoidances, and total response
Van der Borght et al, 2007	- N=80; male C57BL/6 mice<br>- Age: 10 weeks	- Running wheel<br>- 85% CR<br>- 2 weeks	- Exe or sed	- Immunohistochemistry<br><br>- Y maze	- Exe sig. increase pCREB+ cells, DCX+ cells, and DCX+ dendrites<br>- Exe sig. better in maze acquisition, memory retention, and reversal learning
**Nutrition**					
Adams et al, 2008	- N=136; male F344xBN hybrid rats	- CR: 60% of AL	- CR: young, middle aged, old<br>- AL: young, middle aged, old	- MWM<br><br>- Synaptic proteins	- Training: decreased total distance to platform and escape latency in old CR<br>- Higher levels in old CR
Carter et al, 2009	- Male F344xBN hybrid rats	- CR: 60% of AL	- CR: young, middle aged, old, very old<br>- AL: young, middle aged, old, very old<br>- N= 8-12 per group	- ORT<br><br>- MWM	- AL focused more on novel object than CR<br>- Spatial discrimination: CR decreased latency through increased velocity<br>- Proximity to platform: CR less than AL
Fitting et al, 2008	- N= 36; male F344xBN hybrid F1 rats	- CR: 60 % of AL	- CR: old (until 36 months)<br>- AL: young, old	- MWM	- Latency: old CR shorter than old AL<br>- Distance correlate with latency<br>- Speed: no between group difference<br>- Quadrant preference, platform crossing, and centroid: no improvement by CR
Ito et al, 2009	- N= 11; young adult male wistar rats	- 1% taurin (in water) supplementation<br>- one week	- Taurin or control<br>- N= 6 or 5	- MWM	- No diff. between the two groups
Kuhla et al, 2013	- N= 70; female C57BL/6 mice	- CR: 60% of AL	- CR: 4, 20, 74 weeks, late onset (age)<br>- AL: 4, 20, 74 weeks (age)<br>- N= 10 per group	- MWM	- Latency: 74 weeks CR show better learning than 74 weeks AL<br>- Speed: no diff.<br>- Platform crossing: 74 weeks CR better than 74 weeks AL

Lee et al, 2000	- N= 48; male Sprague-Dawley rats	- CR: food on alternate days<br>- 3 months	- CR or AL<br>- N= 24 per group	- Neurogenesis<br><br>- BDNF mRNA levels	- CR: sig. more percentage survival of newly generated cells<br>- CR increase of BDNF mRNA levels in pyramidal neurons in the hippocampus and in neurons throughout the cortex
Lee et al, 2002	- N= 56; adult male C57BL/6 mice	- CR: food on alternating days<br>- 3 months	- CR or AL<br>- N= 28 per group	- Neurogenesis<br><br><br><br><br><br>- Neutrophins	- CR sig. more BrdU+ cells<br>- Co-expression of NeuN and MAP2ab in granule cell layer and GFAP in molecular layer (at 4 weeks)<br>- CR sig. BDNF mRNA increase in pyramidal neurons in the hippocampus<br>- CR sig. increase of NT-3 in dentate granule cells in hippocampus
Markowska et al, 1999	- N= ca. 120; male fisher-344 rats	- CR: 60% of AL	- CR: 6, 12, 18, 24 months (age)<br>- AL: 6, 12, 18, 24 months (age)<br>- N= 10-20 per group	- MWM 1 (normal), MWM 2 (platform changed every session)	- Longer distance in MWM1 for CR
Young et al, 2007	- N= 33; young male long evans rats	- Niacin supplementation: 4g added nicotinamide/ kg<br>- 15 days	- Niacin or control	- MWM<br><br><br><br>- NAD+ and cADPR concentration	- Control group performed better on day three<br>- Niacin and control did not differ in the number of target platform crossing<br>- Niacin: higher NAD+ and cADPR concentrations

Abbreviations: AL = Ad libitum, BDNF = Brain-derived neurotrophic factor, BrdU = Bromodeoxyuridine, cADPR = Cyclic-adenosine diphosphate-ribose, CR = Calorie restriction, DCX = Doublecortin, EE = Enriched environment, Exe = Exercise, GFAP = Glial fibrillary acid protein, MAP = Mature neuron-specific protein, mRNA = Messenger ribonucleic acid, MWM = Morris Water Maze, NAD = Nicotinamide adenine dinucleotide, NeuN = Neuronal nuclear, NT-3 = Neuriphin-3, ORT = Object Recognition Test, pCREB = Phosphorylated cyclic adenosine monophosphate response element-binding protein, RW = Running wheel, Sed = Sedentary, TM = Treadmill
