# Supplementary material for: Effects of Physical Exercise Combined with Nutritional Supplements on Aging Brain Related Structures and Functions: A Systematic Review
Source: Front Aging Neurosci. 2016 Jul 6;8:161. doi: 10.3389/fnagi.2016.00161 (PMC4933713; doi:10.3389/fnagi.2016.00161)
Supplement: Supplementary file 5 [file Table5.pdf]

Supplementary Material

Effects of physical exercise combined with nutritional supplements on aging brain related structures and functions: A systematic review

Alexandra Schättin[*+], Kilian Baur[2+], Jan Stutz[1], Peter Wolf[2], Eling D. de Bruin[1]

[1] Department of Health Sciences and Technology, Institute of Human Movement Sciences and Sport, ETH Zürich, HIT J 32, Wolfgang-Pauli-Str. 27, 8093 Zurich, Switzerland

[2] Department of Health Sciences and Technology, Sensory-Motor Systems Lab, ETH Zürich, TAN E 4, Tannenstrasse 1, 8092 Zurich, Switzerland

[+] shared first author

*** Correspondence:** Alexandra Schättin: schaetta@hest.ethz.ch

Supplementary Table

Supplementary table 5. Included human studies for physical exercise or nutritional intervention. The studies are reported by subjects, intervention, groups, outcome measure, and results.

Study	Subjects	Intervention	Groups	Outcomes	Results
**Physical exercise**					
Bastone and Filho, 2004	- N= 36; healthy adults<br>- Mean age: 68.7±3.8 years	- Aerobic exercise and strength exercise<br>- 60min, 2×/week for 6 months	- Exe or no exe (control)<br>- N= 20 each group	- MMSE	- Non exe sig. diminished score

Baum et al, 2003	- N= 20; residents<br>- Mean age: 88 years	- Aerobic exercise<br>- 60min, 3×/week for 6 months	- Exe or recreational therapy, card, painting<br>- N= 11 or 9	- MMSE	- Exe sig. increase while control decrease
Blumenthal et al, 1991	- N= 101; healthy older adults<br>- Age range: 60-83 years	- Aerobic exercise<br>- 45min, 3×/week for 4 months	- Exe (I), Yoga (II), waiting list (III)<br>- N= 33, 34, 34	- Memory testing, perceptual motor function, nonverbal and verbal fluency tests, Stroop colour word test	- Improvements did not correlate with changes in aerobic power
Brown et al, 2009	- N= 154<br>- Age range: 62-95 years	- Strength exercise, balance, coordination, flexibility<br>- 40min, 2×/week for 6 months	- Exe (I), flexibility and relaxation (II), no exe (III)<br>- N= 82, 34, 38	- Fluid intelligence<br><br>- Executive function and memory	- (I) minor improvement, no change for (II)+(III)<br>- No effects
Cassilhas et al, 2007	- N= 62; male<br>- Age range: 65-75 years	- Strength exercise: moderate (50% RM) or high intensity (80% RM)<br>- 60min, 3×/week for 6 months	- Exe moderate (I), exe high (II), no exe (III)<br>- N= 19, 20, 23	- Similarities, digit span, Corsi's back tapping, Toulouse-Pieron's concentration attention test, Rey-Osterrieth complex<br>- Serum IGF-1	- (I) higher delta means than control (III) for all test<br>- (II) sig. better for Rey-Osterrieth complex<br>- (I)+(II) higher concentration than (III)
Colcombe et al, 2004	- N= 29; community dwelling<br>- Mean age: 65.6±5.7 years	- Aerobic exercise (30/40%-60/70% HR)<br>- 40-45min, 3×/week for 6 months	- Exe or stretching/toning	- fMRI<br><br><br><br>- Flanker task	- Exe sig. greater task-related activity in attentional control areas<br>- Exe sig. reduced level of activity in the anterior cortex<br>- Exe showed 11% reduction in behavioural conflict

Colcombe et al, 2006	- N= 59<br>- Age range: 60-79 years	- Aerobic exercise<br>- 60min, 3×/week for 6 months	- Exe or stretching/toning	- MRI	- For exe group:<br>- Large change for frontal lobe<br>- Grey matter increase: anterior cingulate cortex and supplementary motor cortex, right inferior frontal gyrus, left superior temporal gyrus<br>- Increase in anterior white matter
Dustman et al, 1984	- N= 43<br>- Age range: 55-70 years	- Aerobic exercise<br>- 60min, 3×/week for 4 months	- Exe (I), exe control (II), non exe (III)	1) Critical Flicker Fusion, 2) Culture fair intelligence, 3) Digit span, 4) Digit symbol WAIS subtest, 5) Dots estimation, 6) Reaction time, 7) Stroop colour test	- (I) improved in tests 1, 4, 5, 6, and 7<br>- (II) improved in test 5<br>- (III) improved in test 2<br>- (I)+(II) sig improvement for combined score
Ericksen et al, 2011	- N= 120; community dwelling<br>- Age range: 65-67±6 years	- Aerobic exercise (60-75% HR)<br>- 40min, 3×/week for 12 months	- Exe or stretching/toning<br>- N= 60 each group	- MRI (hippocampus)<br><br><br><br><br><br>- Serum BDNF<br><br>- Spatial memory	- Exe sig. increase by ca. 2% and decrease by 1.5% in control group<br>- Sig. increase of anterior hippocampus<br>- Change in VO2max associated with change in volume<br>- Exe: BDNF correlate with change in hippocampus volume<br>- Exe: change in hippocampus volume associated with change in memory performance
Hill et al, 1993	- N= 121<br>- Mean age: 64±3.1 years	- Aerobic exercise (up to 80% HR)<br>- max. 50min, 3-5×/week for 12 months	- Exe or non exe<br>- N= 87 or 34	- WMS logical memory subtest, WAIS-R, crossing off task	- No effects for exe group

Kamijo et al, 2009	- N= 12<br>- Age range: 60-74 years	- Aerobic exercise (30 or 50% of VO2max)<br>- 20min, single session	- Baseline values (I), post exercise 30% (II) or 50% (III)	- Modified Flanker task<br><br>- EEG	- (III) sig. shorter reaction time compared to (II)+(I)<br>- (II)+(III) shorter P3 latency compared to (I)
Kramer et al, 1999	- N= 127<br>- Age range: 60-75 years	- Aerobe exercise<br>- for 6 months	- Exe or stretching/toning	- Task switching<br>- Response compatibility<br><br>- Stopping test	- Exe much faster than control<br>- Exe decreased the distractor interference effect<br>- Exe reduced reaction time
Liu-Ambrose et al, 2010	- N= 155<br>- Mean age: 69.6±3 years	- Strength exercise<br>- 60min, 1-2×/week for 12 months	- Exe once (I), exe twice (II), stretching/core strength (III)<br>- N= 52, 54, 49	- Stroop test<br>- TMT A/B and verbal digit forward and backward<br>- MRI	- (I)+(II) sig. improved<br>- No sig. diff. between groups<br><br>- (I)+(II) sig. decrease of whole brain volume
Moul et al, 1995	- N= 30; sedentary<br>- Mean age: 69.1±0.79 years	- Aerobic exercise (up to 65% HR)<br>- Strength exercise<br>- 30-40min, 5×/week for 4 months	- Aerobic exe (I), strength exe (II), stretching (III)<br>- N= 10 each group	Subtests of the RIPA: 1) Immediate memory, 2) Recent memory, 3) Temporal orientation, 4) Problem solving and abstract reasoning, 5) Organization, 6) Auditory processing	- 1, 2, 3, and 4: no diff.<br>- 5 and 6: (I) improved sig.<br>- Total score: (I) improved sig.
Muscari et al, 2010	- N= 120<br>- Mean age: 69.6±2.8 years	- Aerobic exercise<br>- 60min, 3×/week for 12 months	- Exe or suggestions to improve lifestyle<br>- N= 69 or 60	- MMSE	- Exe and control worsened sig.<br>- For control but no exe: temporal orientation, attention and calculation, language

Niemann et al, 2014	- N= 36<br>- Mean age: 68.7±3.8 years	- Aerobic exercise<br>- Coordination training<br>- 45-60min, 3×/week for 12 months	- Exe (I), coordination (II), stretching/ relaxation (III)<br>- N= 14, 13, 9	- Flanker and visual search task<br>- Basal ganglia volume<br><br><br><br>- Association of cognition and BGV	- Accuracy and performance speed: (II) improved in both tasks<br>- No sig. effect for total BGV<br>- (II): increase of globus pallidus and caudate<br>- No direct association
Ozkaya et al, 2005	- N= 44; sedentary<br>- Age range: 60-85 years	- Aerobic exercise (70% HR)<br>- Strength exercise (3 set of 12 repetition for 7 exercise)<br>- 50min, 3×/week for about 2 months	- Aerobic exe (I), strength exe (II), no exe (III)<br>- N= 13, 13, 18	- EEG: N1, N2, P2, P3 latencies and N1P2, P2N2, N2P2 amplitudes	- (II) improved in P2 and N2 (Fz,Cz) and N1 (Fz) compared to (III)<br>- (I) improved in P2 and N2 (Fz) and N2 (Cz) compared to (III)<br>- (II) improved in P2 and N2 (Fz, Cz) compared to (I)<br>- Amplitude: no diff. but (II) higher change than (I)+(III)
Perrig-Chiello et al, 1998	- N= 46<br>- Mean age: 73.2 years	- Strength exercise<br>- 1×/week for about 2 months	- Exe or no exe<br>- N= 23 each group	- Word list and digit symbol	- *Short term effect (after 2 months):*<br>Free recall: no diff. between groups but exe improved sig. in delayed free recall<br>Recognition: no diff. between groups but exe improved sig.<br>- *Long term effects (after 1 years):* sig. effect in exe for memory performance

Ruscheweyh et al., 2011	- N= 62; community dwelling<br>- Mean age: 60.2±6.6 years	- Aerobic exercise (50-60% of maximum)<br>- Gymnastics (stretching, limbering, toning)<br>- 50min, 3-5×/week for 6 months	- Aerobic exercise (I), gymnastics (II), non exe (III)<br>- N= 20, 21, 21	- Auditory verbal learning test<br><br><br><br>- MRI<br><br><br><br>- Blood levels: neutrophin and catecholamine	- No group × time interaction<br>- Sig. positive association between changes in physical activity and change in memory<br>- Sig. correlation between change in local grey matter volume and change in physical activity level<br>- No group × time interaction<br>- No association with change in physical activity and change in blood levels
Voelcker-Rehage and Staudinger, 2011	- N= 44<br>- Mean age: 69.64±3.84 years	- Aerobic exercise<br>- Coordination training<br>- 60min, 3×/week for 12 months	- Exe (I), coordination (II), non exe (III)<br>- N= 17, 16, 11	- Modified flanker task and visual search task<br><br><br><br><br><br><br>- fMRI during flanker task	- Accuracy: (I)+(II) improved sig for flanker task, (II) improved sig. and (I) improved marginally sig. for visual search task<br>- Performance speed: (I) improved marginally sig. for flanker task, (II) improved sig. for visual search task<br>- (I)+(II): decreased activation in prefrontal areas<br>- (I): association with sensorimotor network<br>- (II): association with visual spatial network
Zlomanczuk et al, 2006	- N= 41; female<br>- Age range: 57-72	- Aerobic and strength exercise<br>- 45 min, 3×/week for 3 months	- Exe or non exe<br>- N= 33 or 8	- Face/name association<br>- Stroop test	- Exe sig. better than non exe<br>- No diff. between the groups

**Nutrition**					
Cockle et al, 2000	- N= 139<br>- Age range: 60-83 years	- Multivitamin (vitamin A, B12, C, E, thiamine, riboflavin, pyridoxine, biotin, nicotinamide)<br>- 1 RDA: Vitamin A and 10 RDA: others for 24 weeks	- Multivitamin or placebo	- Choice reaction time<br><br>- Critical Flicker Fusion, Sternberger memory scanning test, Word scan task	- Multivitamin fewer decline in total reaction time<br>- No effects
Deijen et al, 1992	- N= 82; male<br>- Age range: 70-79 years	- Vitamin B6<br>- 20mg for 12 weeks	- Vitamin B6 or placebo	- Sperling whole report task, short term verbal memory, long term verbal memory, long term memory storage, long term visual memory, Vienna determination unit	- Short term memory: vitamin group performed better, no multivariate diff. between groups<br>- Long term memory: no diff. between groups for visual part, sig. decrease for placebo, vitamin group remained unchanged for verbal part<br>- Forget score: vitamin group got sig. smaller
Grodstein et al, 2007	- N = 4054 (long part)<br>- N= 1904 (short part)<br>- Age: 73 years	- Beta carotene<br>- 50mg for 1 year or 18 years	- Beta carotene or placebo	- Telephone interview: MMSE, east Boston memory test, word list, category fluency task	- Short term effect: no evidence for cognitive benefits<br>- Long term effect: vitamin group performed sig. better on global score and verbal memory
Grodstein et al, 2013	- N= 5947<br>- Mean age: 71.6 years	- Beta carotene, vitamin E, C or multivitamin<br>- Average length: 8.5 years	- Multivitamin or placebo<br>- N= 2980 or 2967	- Telephone interview: MMSE, east Boston memory test, word list, category fluency task	- No diff. between mean global composite score or any of the tests

Kang et al, 2006	- N= 6377; women	- Vitamin E<br>- 600 IU for 10 years	- Vitamin E or placebo<br>- N= 3184 or 3193	- Telephone interview: MMSE, east Boston memory test, word list, category fluency task	- No diff between groups for global score and for verbal memory
Kelly et al, 2013	- N= 12<br>- Mean age: 64±4 (m), 63±2 (f) years	- NO3 rich beet juice<br>- 140ml/day for 2.5 days	- NO3 or placebo	- Serial subtractions, rapid visual information processing, number recall<br>- 1H MRS (left frontal white matter/occipital parietal grey matter)	- No sig. diff.<br>- No diff. in brain metabolite concentrations (N-acetyl aspartate, creatine, choline, myo-inositol)<br>- No diff. in apparent diffusion coefficients
Macpherson et al, 2012	- N= 56; community dwelling females<br>- Age range: 64-82 years	- Daily multivitamin, antioxidant and mineral formula with added herbal and antioxidant plant extracts<br>- For 16 weeks	- Supplement or placebo	- Simple/complex reaction time, recognition memory, Stroop, spatial working memory, contextual recognition memory, word list<br>- Primary outcomes: memory and attention composite measure	- Only working memory was sig. (decrease in response time for multivitamin group)<br>- Verbal memory: no treatment effect<br>- No sig diff. for both composite scores<br>- memory composite score and all single memory scores were better for multivitamin
Malaguarnera et al, 2007	- N= 70<br>- Age range: 100-106 years	- L-Carnitin<br>- 2g for 6 months	- L-Carnitin or placebo	- MMSE	- Sig. Increase of L-Carnitin group
McMorris et al, 2007	- N= 32<br>-Mean age: 76.4±8.48 years	- Creatine<br>- 5g of creatine monohydrate, first week: placebo, second week: creatine	- Creatine or placebo<br>- n= 15 or 17	- Random number generation test<br>- Forward and backward verbal recall test, spatial recall test, LTM test	- No group diff.<br>- Creatine group improved sig compared to placebo

McNeill et al, 2007	- N= 910; community dwelling<br>- Age range: 71-72 years	- Vitamin A, C, D, E, thiamin, riboflavin, niacin, pantothenic acid, pyridoxine, B12, folic acid, iron, iodine, copper, zinc, manganese<br>- for 1 year	- Supplement or placebo<br>- N= 456 or 454	- Digit span forward test and verbal fluency test	- No evidence for a diff. in the overall change between the 2 groups in either of the 2 tests<br>- over 75 years and those at risk for nutritional deficiencies: no evidence (weak evidence for verbal fluency test)
Presley et al, 2011	- N= 16<br>- Mean age: 74.7±6.9 years	- High nitrate diet<br>- For 2 days	- High nitrate or low nitrate diet	- Brain imaging: CBF	- No global perfusion diff.<br>- Increased CBF within the subcortical and deep white matter of the frontal lobe
Rossom et al, 2012	- N= 4143<br>- Age range: 65-80 years	- Vitamin D3: 400IU<br>- Calcium: 1 g<br>- Average length: 8 years (MMSE) or 5 years (cognitive tests)	- Supplement or placebo	- Modified MMSE<br><br>- Attention and working memory, verbal knowledge, spatial ability, verbal fluency, verbal memory, short-term figural memory, and fine motor speed	- No diff. at each year of the 8 years<br>- No sig. diff. in any domain specific cognitive scores
Smith et al, 1999	- N= 185<br>- Mean age: 65-66±0.5 years	- Antioxidants: 12mg beta carotene, 400mg alpha tocopherol, 500mg ascorbic acid<br>- For 1 year	- Antioxidant or placebo<br>- N= 93 or 92	- Free recall task, delayed recognition memory task, logical reasoning task, simple reaction time task, repeated-digits vigilance task, focused attention task, categorical search task	- Analysis of variance comparing the placebo and vitamin groups identified only 4 sig diff. in 117 analyses<br>- Majority of the analyses showed no sig. relationship between vitamin status and performance

Smith et al, 1999	- N= 205<br>- Mean age: 67±1 years	- Antioxidants: 12mg beta carotene, 400mg alpha tocopherol, 500mg ascorbic acid<br>- For 1 year	- Antioxidant or placebo	- New Adult reading test and cognitive failures questionnaire	- Antioxidant revealed little effect on mood or cognitive function<br>- Improvement in mood and cognition for subjects with high changes in vitamin C but not for other vitamins
Summers et al, 2010	- N= 113<br>- Mean age:60-63±7 years	- Antioxidant: vitamin, lipids, amino, acids, minerals<br>- Six gels per day for 4 months	- Antioxidant or placebo<br>- 59 or 54	- MMSE<br><br>- An expanded 50-item names-learning paired association test<br>- 20 word free recall test of short term memory	- Antioxidant from 29.3 to 29.7 and placebo from 29.1 to 29.3<br>- Antioxidant group sig. higher compared to the placebo group<br><br>- Sig change in the antioxidant group compared to placebo group
Szczesniak et al, 2014	- N= 51<br>- Mean age: 80.8±7.8 years	- Anserine and carnosine supplement<br>- 2.5 per day for 13 weeks	- Supplement or placebo	- MMSE<br><br><br><br>- STMS	- Sig improvement in supplement group, none in placebo<br>- Sig improved in people aged 81-94 but not in 65-80<br>- Sig improvement in supplement group (in 3 sub-scores), none in placebo
Wolters et al, 2005	- N= 220<br>- Age range: 60-74 years	- Multivitamin: vitamin A, B1, B2, B5, B6, B12, C, E, H, magnesium, niacin, folic acid, selenium<br>- For 6 months	- Multivitamin or placebo<br>- N= 111 or 109	- Subtest of WAIS-III, Kurztest für allgemeine Intelligenz, subtest of the Berliner Amnesie Test	- Same improvements in both groups

Yaffe et al, 2004	- N= 2166<br>- Mean age: 63 years	- Antioxidant: vitamin C, E, beta carotene plus zinc and/or copper<br>- For average of 7 years	- Antioxidant: (zinc) (I), (copper) (II), (zinc and copper) (III) or placebo (VI)	- Modified MMSE, animal category, letter fluency, WMS-R, immediate recall, word list, Buschke selective reminding test, digit backwards	- No sig diff. in all groups
Yasuno et al, 2012	- N= 41<br>- mean age: 72.7±4.8 years	- Fish oil: 290mg EPA, 203mg DHA<br>- Lycopene: 84 mg<br>- Gingko biloba extract: 240mg<br>- For 3 years	- Supplement or placebo<br>- N= 41 or 622	- Japanese version of set-dependent activity<br>- category cued recall test<br>- category fluency test, abstract<br>- similarities subtest of WAIS-R<br>- composite score	- Supplement group was better after 3 years, no group changed sig.<br>- Sig increase in both groups, higher in supplement group<br>- No change for supplement group, placebo sig. decreased<br>- No change for both groups<br>- Sig increase in supplement group, no change for placebo

Abbreviations: BDNF = Brain-derived neurotrophic factor, BGV = Basal ganglia volume, CBF = Cerebral blood flow, DHA = Docosahexaenoic acid, EEG = Electroencephalography, EPA = Eicosapentaenoic acid, Exe = Exercise, fMRI = Functional magnetic resonance imaging, HR = Heart rate, IGF-1 = Insulin like growth factor 1, IU = International unit, LTM = Long term memory, MMSE = Mini Mental Status Examination, MRI = Magnetic resonance imaging, MRS = Magnetic resonance spectroscopy, NO3 = Nitrate, RDA = Recommended dietary allowance, RIPA = Ross Information Processing Assessment, RM = Repetition maximum, STMS = Short Test of Mental Status, TMT A/B = Trail Making Test A/B, WAIS = Wechsler Adult intelligence Scale, WMS = Wechsler Memory Scale
